# Hierarchical design of pseudosymmetric protein nanocages

**DOI:** 10.1038/s41586-024-08360-6

**Published:** 2024-12-18

**Authors:** Quinton M. Dowling, Young-Jun Park, Chelsea N. Fries, Neil C. Gerstenmaier, Sebastian Ols, Erin C. Yang, Adam J. Wargacki, Annie Dosey, Yang Hsia, Rashmi Ravichandran, Carl D. Walkey, Anika L. Burrell, David Veesler, David Baker, Neil P. King

**Affiliations:** 1 Department of Bioengineering, University of Washington, Seattle, WA USA; 2 Institute for Protein Design, University of Washington, Seattle, WA USA; 3 Department of Biochemistry, University of Washington, Seattle, WA USA; 4 Howard Hughes Medical Institute, Seattle, WA USA

**Keywords:** Protein design, Cryoelectron microscopy, Bioinspired materials

## Abstract

Discrete protein assemblies ranging from hundreds of kilodaltons to hundreds of megadaltons in size are a ubiquitous feature of biological systems and perform highly specialized functions^1,2^. Despite remarkable recent progress in accurately designing new self-assembling proteins, the size and complexity of these assemblies has been limited by a reliance on strict symmetry^3^. Here, inspired by the pseudosymmetry observed in bacterial microcompartments and viral capsids, we developed a hierarchical computational method for designing large pseudosymmetric self-assembling protein nanomaterials. We computationally designed pseudosymmetric heterooligomeric components and used them to create discrete, cage-like protein assemblies with icosahedral symmetry containing 240, 540 and 960 subunits. At 49, 71 and 96 nm diameter, these nanocages are the largest bounded computationally designed protein assemblies generated to date. More broadly, by moving beyond strict symmetry, our work substantially broadens the variety of self-assembling protein architectures that are accessible through design.

## Abstract (web summary)

A new computational method for design of pseudosymmetric self-assembling protein nanomaterials has resulted in purification of cage-like protein assemblies containing 960 subunits with a diameter of 96 nm.

## Main

Self-assembling protein complexes are ubiquitous structures that are foundational to living systems. These structures vary in size from a few nanometres to micrometre-sized viral capsids and perform a wide variety of structural and biochemical functions^1,2^. The information that drives assembly of these complexes is encoded in their amino acid sequences and functionally takes the form of the structures of individual protein subunits and the interactions between them. The unique properties of self-assembling proteins have been exploited for applications in drug delivery, enzyme encapsulation and vaccines^4–7^. However, relying on naturally occurring assemblies constrains the engineer to existing sizes, shapes and levels of complexity. Methods for generating new self-assembling proteins render additional classes of structures and functions accessible, enabling these properties to be tailored to specific applications^8^.

Advances in methods for controlling or designing the way protein subunits interact has led to an explosion of new designed assemblies in recent years, particularly those with finite, point-group symmetries^9^ (that is, oligomers, nanocages and capsids). Engineered nanocages and capsids have been generated by computational protein design^10–14^, rational design^15^, genetic fusion and domain swapping^16–19^, metal coordination^20–22^ and laboratory evolution^23,24^. Each of these methods has a characteristic level of precision and predictive capacity. Computational docking and protein–protein interface design stands out for its ability to consistently create new protein complexes with atomic-level accuracy, although with a relatively modest success rate owing to the unique challenge posed by each interface design problem. Nevertheless, computationally designed protein nanocages have been engineered to encapsulate small molecules, nucleic acids and other polymers^25,26^; evolved for improved cargo packaging and extended in vivo half-life^25^; applied to enhance receptor-mediated signalling and virus neutralization^27,28^; and used as scaffolds for structure determination^29^, multi-enzyme co-localization^30^ and multivalent antigen presentation^31–34^, including in multiple vaccines currently in clinical development^34,35^ or licensed for use in humans^36,37^. Further development of computational methods will give rise to designed protein nanomaterials of continually increasing sophistication, leading to improved performance in these applications and making additional applications possible.

Design methods reported to date have relied on the use of strict symmetry and pre-existing oligomeric building blocks to reduce the number of new interfaces that must be designed^3,38^. Although this approach yields access to a handful of finite (that is, bounded) symmetric architectures that require only a single designed interface^39^, it nevertheless places a severe constraint on the architectures that are accessible to design and their size and complexity. The largest and most complex structures designed using this approach comprise 120 subunits and have strict icosahedral symmetry, featuring a single copy of each of two subunits in the icosahedral asymmetric unit^11,32^. Developing methods for breaking the symmetry of computationally designed protein assemblies is a key next step in developing more sophisticated self-assembling proteins.

Four routes to larger and more sophisticated protein assemblies exist, each of which is observed in naturally occurring self-assembling proteins. First, larger protein subunits could be used as building blocks, with titin providing an extreme example^40^. However, this approach is untenable as a general solution, as limits in protein translation, folding, stability and flexibility are quickly encountered^2^. Second, the number of different kinds of subunits in the assembly (or its asymmetric unit (asu)) could be increased by designing new asymmetric interactions between them, as observed in multi-subunit molecular machines such as RNA polymerases^41^. Although ultimately we expect this approach to become possible, it is currently impractical, as it would compound the low success rates of existing interface design methods. Third, principles of quasi-equivalence could be used to design large assemblies from protein subunits that adopt subtly different conformations depending on their local symmetry environment, a phenomenon commonly found in icosahedral virus capsids^23,42,43^. However, current computational protein design methods lack the precision required to reliably encode in a single amino acid sequence the multiple subtly different backbone conformations required to implement this approach. Finally, pseudosymmetry could be used to enable asymmetric functionalization of oligomeric building blocks, opening up new routes to the design of larger assemblies. Pseudosymmetry is also frequently observed in icosahedral virus capsids, where genetically distinct subunits or domains adopt roughly symmetric orientations within oligomeric capsomers^44^. For example, pseudosymmetric trimers in virus capsids may comprise three subunits, each containing two related but slightly distinct domains that result in an (A–B)–(A–B)–(A–B) arrangement with roughly sixfold symmetry at the backbone level^45,46^. Such trimers can be arranged in hexagonal lattices that form the facets of very large icosahedral assemblies; the A and B domains form the distinct sets of contacts that are necessary to form non-porous lattices. Although designing pseudosymmetric assemblies requires the creation of multiple new protein–protein interfaces, a hierarchical approach in which pseudosymmetric oligomers are designed first and subsequently used as the building blocks of larger pseudosymmetric assemblies would enable the distinct interfaces to be designed and validated individually. This approach avoids compounding the relatively high failure rate of interface design and, as we show, permits the design of novel cage-like protein nanomaterials that far exceed the size and complexity of previously designed assemblies.

## Pseudosymmetric heterotrimer design

**Fig. 1:**
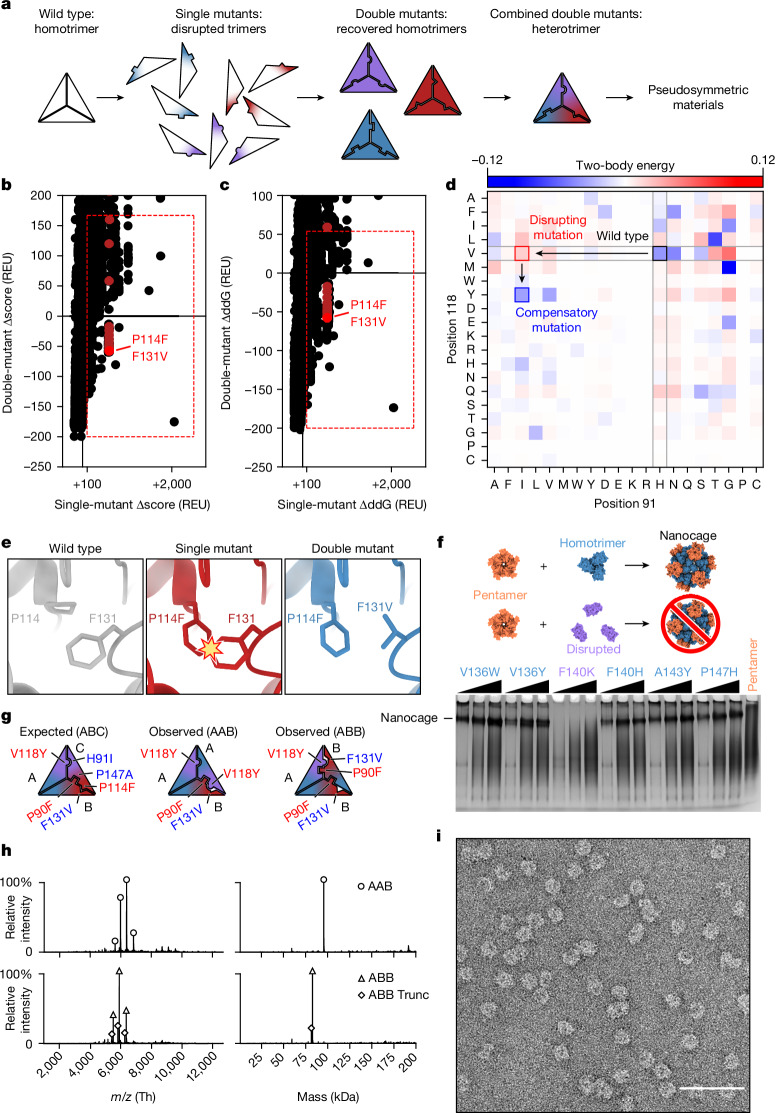
Design and characterization of a pseudosymmetric heterotrimer. **a**, The design protocol starts with a homotrimer to which single trimer-disrupting mutations are introduced, followed by compensatory mutations that rescue trimer assembly. Sets of orthogonal mutations (depicted as red, purple and blue) are combined to generate a heterotrimer that can then be used as a component in pseudosymmetric materials. **b**,**c**, Calculated changes in Rosetta score (Δscore) (**b**) and predicted trimerization energy (ΔddG) (**c**) upon mutation were used to evaluate single mutants (horizontal axis) and double mutants (vertical axis). The red boxes enclose mutants that met selection criteria for further evaluation, and mutant pairs containing P114F, shown in **e**, are highlighted in red. REU, Rosetta energy units. **d**, Possible mutations were also evaluated by their co-evolution coupling matrix. Desirable mutations are those for which the single-mutant–wild-type pair is observed less frequently than expected (red; H91I/V118) and the double-mutant pair is observed more frequently than expected (blue; H91I/V118Y). **e**, An example of a productive mutant pair in which the wild-type residue F131 clashes with the mutant residue P114F and the second mutation F131V resolves the clash. **f**, Disruption of trimer geometry was assayed by assembling mutant I53-50A trimers in clarified *E. coli* lysates with purified I53-50B pentamer and evaluating the presence or absence of I53-50 nanocages by native PAGE. Here, F140K was identified as a disrupting single mutation. Black wedges indicate increasing pentamer concentration in the assembly reaction. Data are representative of three independent experiments. For gel source data, see Supplementary Fig. 1. **g**, Diagram of the expected ABC heterotrimer and the observed AAB and ABB heterotrimers. Disrupting mutations are labelled in red and compensatory mutations are in blue. **h**, Native mass spectrometry of AAB-enriched (top) and ABB-enriched (bottom) heterotrimer fractions purified by IMAC and SEC. ABB Trunc refers to a truncation product of the A chain in which the N-terminal ten residues of the protein were missing. **i**, Assembly of I53-50-like nanocages using an AAB/ABB mixture of I53-50A heterotrimer was verified by negative-stain electron microscopy. Scale bar, 100 nm.

**Extended Data Fig. 1:**
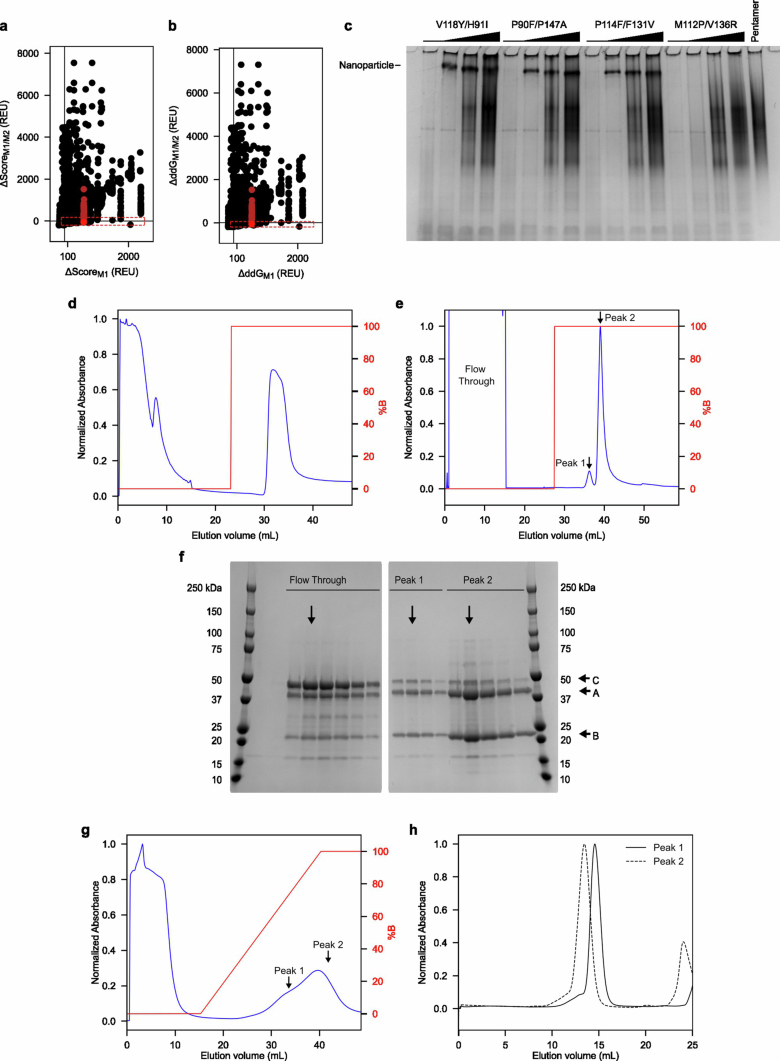
“ABC” design and purification and characterization of “ABC” tricistronic and “AB” bicistronic constructs. **a**, ΔddG filter metric. **b**, ΔScore metric. Dark red points correspond to the single mutation P114F. The bright red point corresponds to the double mutant P114F/F131V. The red dotted boxes represent cutoffs used to select mutants for testing. **c**, Recovery of trimer geometry was assayed by assembling double mutant I53-50A trimers in clarified *E. coli* lysates with purified I53-50B pentamer and evaluating the presence or absence of I53-50 nanocages by native PAGE. Black wedges indicate increasing pentamer concentration in each series of assembly reactions. For gel source data, see Supplementary Fig. 2. **d**, The ABC heterotrimer was purified by IMAC with a step elution followed by **e**, StrepTrap purification. The A chain contained a hexa-histidine and SUMO tag, the B chain contained a Strep tag, and the C chain contained sfGFP and avi tags. The eluate of this two-step purification method should therefore only contain trimers that include both the A and B chains. An optimal result would be equimolar amounts of the A, B, and C chains. **f**, SDS-PAGE of the StrepTrap purification revealed that the eluate contained an excess of the A and B chains and less of the C chain. For gel source data, see Supplementary Fig. 3. To test the ability of the A and B chains only to assemble into heterotrimers, we expressed an AB bicistronic gene and **g**, purified the resulting proteins by IMAC with a gradient elution. Two broad and overlapping peaks were observed. The leading half of the first peak and trailing half of the second peak were collected and **h**, further purified by SEC. Peak 2 has a lower retention volume than peak 1, suggesting a difference in molecular weight. These results are consistent with assembly of an ABB heterotrimer (earlier IMAC elution, later SEC elution) and an AAB heterotrimer (later IMAC elution, earlier SEC elution). We confirmed this interpretation by native mass spectrometry (Fig. 1g).

We started our pseudosymmetric design with a homotrimeric aldolase from the hyperthermophilic bacterium *Thermotoga maritima* that is remarkably stable and tolerant of modification (Protein Data Bank (PDB) ID 1WA3; ref. ^47^). This trimer has previously been used to design multiple one- and two-component protein assemblies^11,14^, which as we show below, makes possible the re-use of these previously designed interfaces in the creation of large pseudosymmetric assemblies. We set out to identify the minimum set of mutations necessary to drive formation of a pseudosymmetric heterotrimer. We used two methods to identify individual mutations predicted to disrupt—as well as compensatory mutations predicted to restore—homotrimer stability, reasoning that combining sets of such mutations across three variants of the trimer subunit could yield pseudosymmetric heterooligomers (Fig. 1a). First, the energetic effects of all possible single and pairwise mutations in 98 contacting residue pairs over 36 positions in the 1WA3 homotrimer interface were evaluated using Rosetta. Ninety-six unique individual mutations increased the predicted homotrimerization energy (ddG) or Rosetta score by more than 100 Rosetta energy units, suggesting that they may disrupt the wild-type homotrimeric interface (Extended Data Fig. 1a,b). Only a subset of these had compensatory mutations that brought the normalized total score or normalized ddG close to zero; these were considered further (Fig. 1b,c, red boxes). Second, because 1WA3 is a naturally occurring protein, we also used bioinformatics to guide our mutant and double-mutant selection. Using GREMLIN^48,49^, we inspected the coupling matrices of highly co-evolving residues at the trimer interface to identify low-frequency single mutations (for example, H91I; PDB ID 1WA3 numbering) with high-frequency compensatory mutations (for example, V118Y) (Fig. 1d). As expected, many of the predicted disrupting mutations identified by both methods were mutations to bulky hydrophobic residues (Fig. 1e). Models of those single mutants were visually inspected and then paired with the best-scoring double mutant. In total, mutations from 76 mutant pairs were selected for experimental screening (Extended Data Table 1).

First, the ability of each single mutation to disrupt trimer formation was screened in a lysate-based assay by evaluating its effect on the assembly of I53-50, a previously reported two-component nanocage^11^ comprising a trimeric component (I53-50A) derived from 1WA3 and a pentameric component (I53-50B) derived from a bacterial lumazine synthase^50^ (PDB ID 2OBX). When I53-50A and I53-50B are mixed, the two components spontaneously self-assemble to form a 120-subunit complex. Clarified lysates from *Escherichia coli* that express the I53-50A mutants were mixed with purified I53-50B pentamer at three different pentamer concentrations and analysed by native (non-denaturing) PAGE (Fig. 1f). The 1WA3 trimer proved remarkably plastic: only 3 out of the 82 single mutants tested did not yield a band corresponding to the assembled I53-50 nanocage, indicating that these either prevented soluble expression of the trimer variant or altered its geometry so that it was no longer assembly-competent. Mutations that prevented nanocage formation were then combined with their compensatory mutation to determine whether the combination restored the ability to form I53-50 nanocages (Extended Data Fig. 1c). Through these analyses, three pairs of functional disrupting and compensatory mutations were identified: H91I/V118Y, P90F/P147A and P114F/F131V.

We initially set out to generate an ‘ABC’ heterotrimer, in which each subunit has a different amino acid sequence, by combining the three mutant pairs in a tricistronic expression construct (all novel amino acid sequences provided in Supplementary Table 1). We introduced one of the homotrimer-disrupting mutations into each subunit: V118Y into A, P90F into B and P114F into C (Fig. 1g). The disrupting mutations in the A and C chains clashed with the neighbouring subunit in a ‘clockwise’ direction, whereas P90F in the B chain clashed with its ‘anticlockwise’ neighbour. As a result, the three compensatory mutations were added to the B (F131V) and C (H91I and P147A) chains. This generated two new interfaces predicted to be orthogonal to the wild-type interface, in principle providing the three interfaces required to form a heterotrimer. However, when we co-expressed the three proteins and purified them by immobilized metal affinity chromatography (IMAC) and StrepTrap chromatography, SDS–PAGE analysis suggested the presence of trimers comprising predominantly a mixture of the A and B subunits, with little of the C subunit (Extended Data Fig. 1d–f). To better understand this off-target species, we expressed a bicistronic gene containing only the A and B subunits. We purified the resulting protein and identified two distinct trimeric assemblies by native mass spectrometry: a trimer comprising one copy of the A chain and two copies of the B chain (‘ABB’), as well as a trimer comprising two copies of the A chain and one copy of the B chain (‘AAB’) (Fig. 1h and Extended Data Fig. 1g,h). Although initially unexpected, we suspected that the remarkable plasticity of the 1WA3 trimer allowed it to tolerate the disrupting mutations in the A and B chains when these were combined with the compensatory mutation F131V, a suspicion that was borne out in later structural studies. Although not the intended ABC heterotrimer, we realized that these heterotrimers were probably pseudosymmetric and, as we describe below, could provide a simple route to designing large pseudosymmetric materials. To confirm that symmetry was preserved at the backbone level—a prerequisite for our hierarchical design approach—we determined whether the heterotrimer mixture was assembly-competent by purifying and incubating it in a 1:1 molar ratio with I53-50B pentamer. Assemblies were purified by size-exclusion chromatography (SEC) and nanocages with the known I53-50 morphology^11^ were observed by negative-stain electron microscopy (Fig. 1i).

## Design of a 240-subunit nanocage

**Fig. 2:**
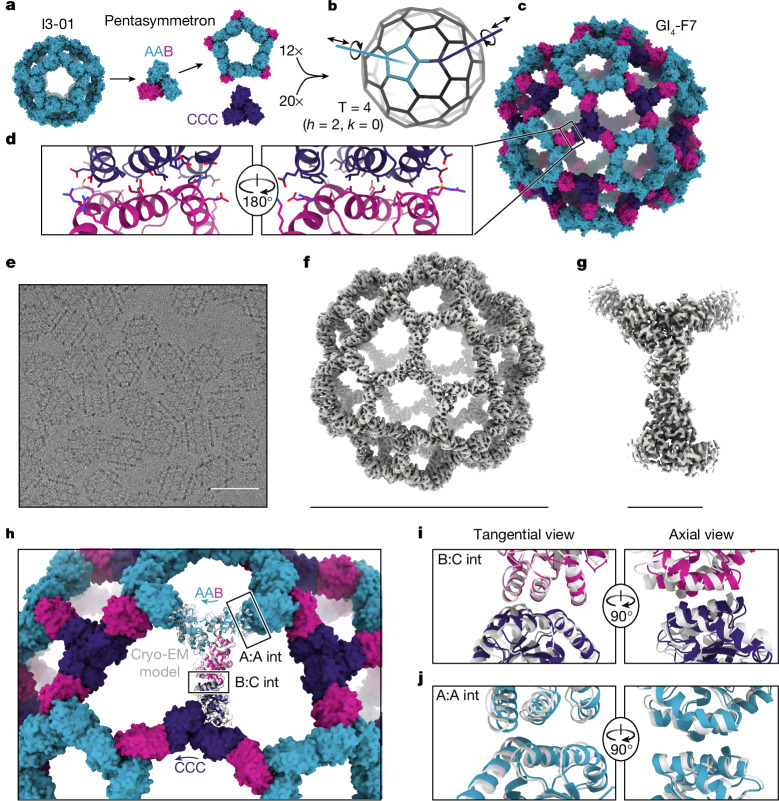
Design and characterization of the 240-subunit GI_4_-F7 nanocage. **a**, Schematic of pentasymmetron generation from I3-01 and the AAB heterotrimer. The A (cyan) subunits in the pentasymmetron retain the two-fold symmetric I3-01 nanocage interface, whereas the B (magenta) subunits are available for docking. **b**, Docking the pentasymmetron as a rigid body against CCC homotrimers (purple) yields 240-subunit, T = 4 assemblies. Translational and rotational degrees of freedom for the pentasymmetron and homotrimer components are indicated. **c**, A design model of GI_4_-F7. **d**, Detail of the computationally designed interface between the B and C subunits of GI_4_-F7 design model. **e**, Cryo-EM micrograph of assembled GI_4_-F7 nanocages embedded in vitreous ice. Scale bar, 50 nm **f**, The 4.4 Å resolution density map of the entire GI_4_-F7 nanocage. Scale bar, 49 nm. **g**, The 3.1 Å resolution density map from an asu obtained via symmetry-expansion and local refinement. Scale bar, 7.4 nm. **h**, Comparison of the cryo-EM structure derived from local refinement (grey ribbon) with the computational design model (coloured ribbons), aligned using a single copy of the asu. Arrows indicate rigid-body deviations of the pentasymmetron (cyan) and CCC homotrimer (purple). Int, interface. **i**,**j**, Detail of the rigid-body deviations from the design model at the B–C interface (**i**) and the A–A (I3-01) interface (**j**). In **j**, two neighbouring copies of the AAB heterotrimer from the full nanocage reconstruction and the design model were aligned.

**Extended Data Fig. 2:**
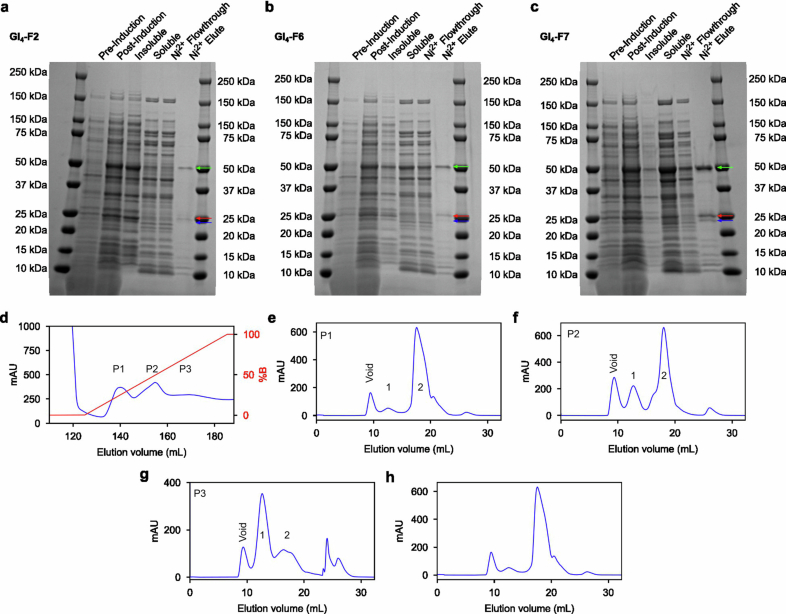
Screening of GI_4_ designs and in vitro assembly of GI_4_-F7 from purified components. Expression and screening by SDS-PAGE for GI_4_ designs **a**, GI_4_-F2, **b**, GI_4_-F6 and **c**, GI_4_-F7. Bands for chains A (green arrow), B (blue arrow), and C (red) arrow are indicated. The presence of all three bands in the Ni^2+^ Elute lanes of GI_4_-F6 and GI_4_-F7 indicates interactions between the A, B, and C chains. For gel source data, see Supplementary Fig. 4. **d**, HisTrap elution chromatogram of AB bicistronic expression. Blue, absorbance at 280 nm; red, gradient elution. Peak 1 (P1) is predominantly ABB, P2 is predominantly AAB, and P3 is predominantly the A chain, which assembles into 60-subunit I3-01-like nanocages. **e**, Superdex 200 Increase 10/300 chromatogram of P1 from the HisTrap elution. The first peak following the void volume (1) is predominantly I3-01-like nanocages and (2) is predominantly ABB heterotrimer. **f**, Superdex 200 Increase 10/300 chromatogram of P2 from the HisTrap elution. (1) is predominantly I3-01-like nanocages and (2) is predominantly AAB heterotrimer. **g**, Superdex 200 Increase 10/300 chromatogram of P3 from the HisTrap elution. (1) is predominantly I3-01-like nanocages and (2) is predominantly AAB heterotrimer. **h**, SEC purification of GI_4_-F7 on a Sephacryl S-500 HR 10/300 GL column. Peak 1 contains the assembly while peak 2 is residual homotrimer component.

We then used the pseudosymmetric heterotrimers to design large, pseudosymmetric assemblies with icosahedral symmetry. We had previously used the 1WA3 homotrimer to generate a single-component nanocage with icosahedral symmetry, I3-01, by designing a novel protein–protein interface with two-fold symmetry between the subunits of adjacent trimers^14^. The existence of this interface enabled us to generate a 15-subunit ‘pentasymmetron’ comprising 5 trimers by simply including the I3-01 mutations on the A chains of the AAB heterotrimer (Fig. 2a). Docking this pentasymmetron against C3-symmetric homotrimers (‘CCC’) and designing novel sequences that create favourable interfaces between the B and C chains yielded models of 240-subunit nanocages with icosahedral symmetry (Fig. 2b–d). The Caspar–Klug triangulation (T) number notation^42^ is useful for describing these pseudosymmetric nanocages, although the assignment of subunits to geometric elements is different than in traditional use of the T number in structural virology. In our pseudosymmetric nanocages, trimeric building blocks form wireframe-like structures surrounding roughly pentagonal and hexagonal pores, with each subunit interacting with exactly one other subunit from a different trimer. The original I3-01 nanocage can be thought of as T = 1, with one (A) subunit in the asu, while the pentasymmetron-containing 240-subunit nanocages are T = 4, with four (2×A, 1×B, 1×C) subunits in the asu. In these assemblies *k* = 0, so the T number is equal to *h*^2^, where *h* is a positive integer that represents the number of steps required to traverse from one pentasymmetron to another, each step moving to the next pentagonal or hexagonal pore. Because this is one of the set of equations used to define class I Goldberg polyhedra^51^, we refer to these nanocages using the naming convention GI_T_-X, where G stands for Goldberg, I for icosahedral symmetry, T is used to denote the triangulation number of a particular architecture, and X is a unique identifier for each design. We expressed three initial designs in *E. coli* as tricistronic genes with a 6×His tag on only the C chain, and found that Ni^2+^ beads co-precipitated all three subunits of two of the designs, suggesting assembly (Extended Data Fig. 2a–c). We proceeded with the better expressing and more soluble of the two, GI_4_-F7. To scale up expression of the AAB heterotrimer so that we could explore assembly of GI_4_-F7 in vitro from purified components, we re-cloned it as a bicistronic AB construct with a 6×His tag on the A chain. Upon gradient elution during IMAC, we observed three peaks corresponding to an ABB-rich fraction, an AAB-rich fraction and off-target AAA homotrimers that assembled to an I3-01-like nanocage (Extended Data Fig. 2d). We polished the AAB and ABB fractions by SEC, discarding the I3-01-like nanocage fraction (Extended Data Fig. 2e–g). This step removed the I3-01-like assemblies but did not resolve the ABB and AAB trimers. We therefore expected some cross-contamination between those trimer species, as observed in the native mass spectrometry data (Fig. 1h). In parallel, we purified 6×His-tagged CCC homotrimer—which was also derived from the 1WA3 trimer—by IMAC and SEC.

**Extended Data Fig. 3:**
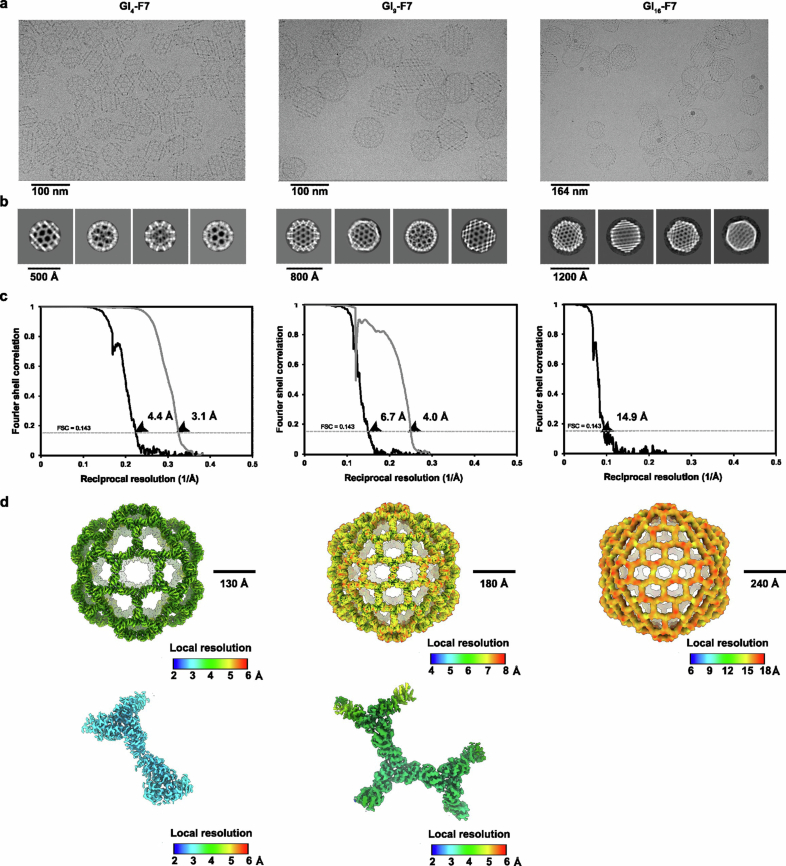
CryoEM data processing. (**a-b**) Representative electron micrographs **a**, and 2D class averages **b**, of GI_4_-F7 (left), GI_9_-F7, (middle) and GI_16_-F7 (right). **c**, Gold-standard Fourier shell correlation curves for the 3D reconstructions of GI_4_-F7 (left), GI_9_-F7 (middle) and GI_16_-F7 (right) (black line) and locally refined asus (gray lines). The 0.143 cutoff is indicated by a horizontal dashed line. (**d-e**) Local resolution maps calculated using cryoSPARC for **d**, the 3D reconstructions of GI_4_-F7 (left) and locally refined asu (left, bottom), GI_9_-F7 (middle) and locally refined asu (middle, bottom), and GI_16_-F7 (right).

**Extended Data Fig. 4:**
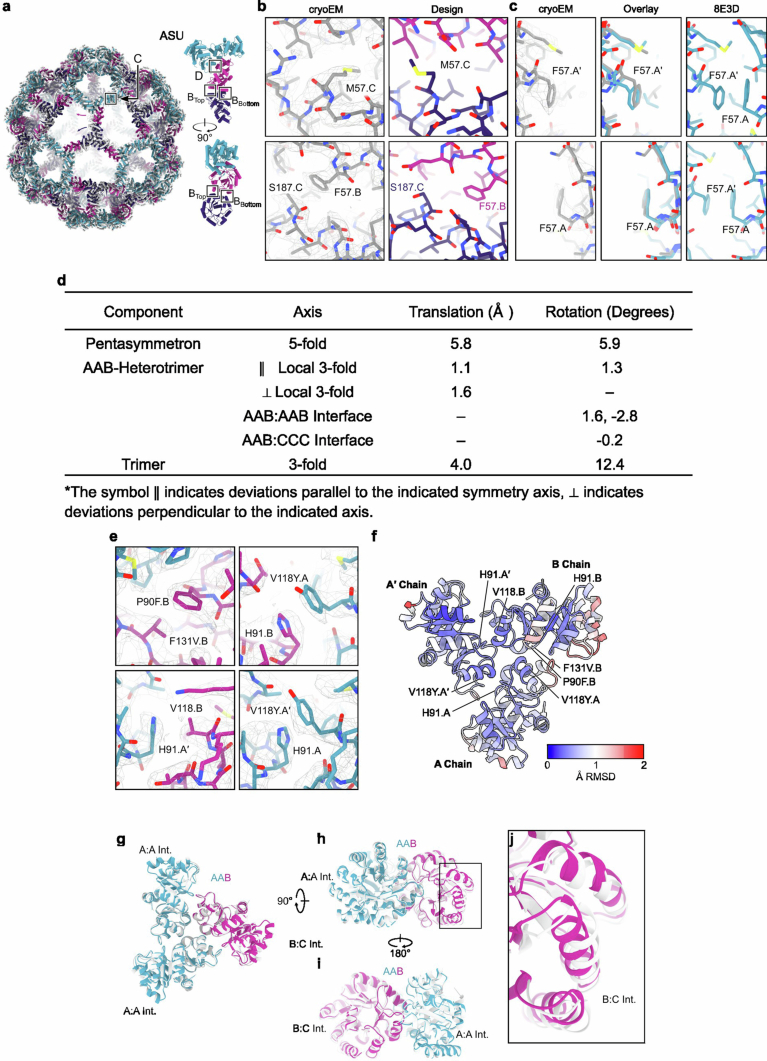
Structural details of GI_4_-F7. **a**, Alignment of the complete cryoEM model to the design model. Major rigid-body DoF deviations are indicated with arrows. Two views of the asu are shown. Approximate locations of each inset (B, C, and D) are indicated. **b**, Comparison between the cryoEM model (left) and design model (right) of the newly designed nanocage (B-C) interface. *Top row*, M57 on the CCC-homotrimer changes rotamer to occupy a void in the interface in the design model. *Bottom row*, F57 on the B chain of the AAB heterotrimer packs against S187 of the CCC homotrimer in the cryoEM model, instead of A190 in the CCC homotrimer as in the design model. **c**, Comparison of the I3-01 (A-A) interface observed in the cryoEM model to a previously published structure (PDB ID 8ED3; ref. 78). *Top row*, slight rigid-body deviations from perfect two-fold symmetry in one copy of the A chain. *Bottom row*, very little deviation from perfect two-fold symmetry. **d**, Details of the density maps in the regions of the pseudosymmetry-generating mutations within the AAB heterotrimer interface. **e**, Pseudosymmetric heterotrimer colored by Cα-RMSD to the design model. The positions of the pseudosymmetry-generating mutations are indicated. **f**, Alignment of the AAB heterotrimer cryoEM model to the design model is viewed from the top, towards the center of the nanocage along the three-fold symmetry axis; **g**, from the side, tangential to the nanocage surface; and **h**, from the other side, tangential to the nanocage surface. The position of the A:A and newly designed B:C interfaces are indicated. **i**, Detail of the B side of the B:C interface, highlighting the most significant deviations from the design model. **j**, Deviations observed in the cryoEM reconstruction of GI_4_-F7 compared to the design model.

We mixed the AAB heterotrimer with an excess of the CCC homotrimer in the presence of detergent and initiated assembly by dialysing overnight into Tris-buffered saline (Methods). The major assembly product was purified by SEC (Extended Data Fig. 2h), and images obtained by cryo-electron microscopy (cryo-EM) of vitrified specimens revealed wireframe structures with large hexagonal pores that closely resembled the design model (Fig. 2e). We determined a single-particle reconstruction of GI_4_-F7 at 4.4 Å resolution applying icosahedral symmetry and a 3.1 Å resolution structure of the 4 chains of the asu (cryo-EM processing details in Extended Data Fig. 3 and Extended Data Table 2). The cryo-EM structure agrees well with the design model, with a Cα root mean-squared deviation (r.m.s.d.) of 9.3 Å across all 240 subunits and 3.0 Å within the asu (Fig. 2f,g and Extended Data Fig. 4). The differences between the cryo-EM structure and design model are mostly accounted for by slight rigid-body deviations allowed by the limited degrees of freedom of the oligomeric building blocks in this symmetric architecture (Extended Data Fig. 4a). The main rigid-body deviation is a 5.9° clockwise rotation of the pentasymmetron, accompanied by a 5.8 Å translation away from the origin (Fig. 2h). The CCC homotrimer compensates by rotating 12.4° and translating 4.0 Å, resulting in only slight local shifts relative to the design model (2.1 Å across the B:C subunits; Fig. 2i). Within the pentasymmetron, the degrees of freedom of the AAB heterotrimer are no longer restricted by the strict icosahedral symmetry of I3-01, resulting in a slight deviation from perfect two-fold symmetry between neighbouring A chains (1.4 Å Cα r.m.s.d.; Fig. 2j and Extended Data Fig. 4d). In addition to these slight rigid-body deviations, the 3.1 Å resolution structure of the asu enabled us to visualize the pseudosymmetry-generating mutations in the A and B subunits. As suspected, we observed backbone and sidechain rearrangements within each protomer that explained how the V118Y and P90F disrupting mutations were tolerated in the AAB heterotrimer. Specifically, we saw that the loop containing H91 and the entire preceding helix shifted relative to the design model in all three subunits. This created enough space to accommodate the P90F mutation in chain B and for V118Y in the A subunits to pack against H91 (Extended Data Fig. 4e,f). Additional minor structural deviations were observed within each subunit, primarily in the B:C interface (Extended Data Fig. 4g–j). Overall, the diameter of GI_4_-F7 observed by cryo-EM is within 2% of the design model, establishing that our method is capable of accurately designing pseudosymmetric protein nanomaterials comprising hundreds of subunits.

## Observation of a 540-subunit nanocage

**Fig. 3:**
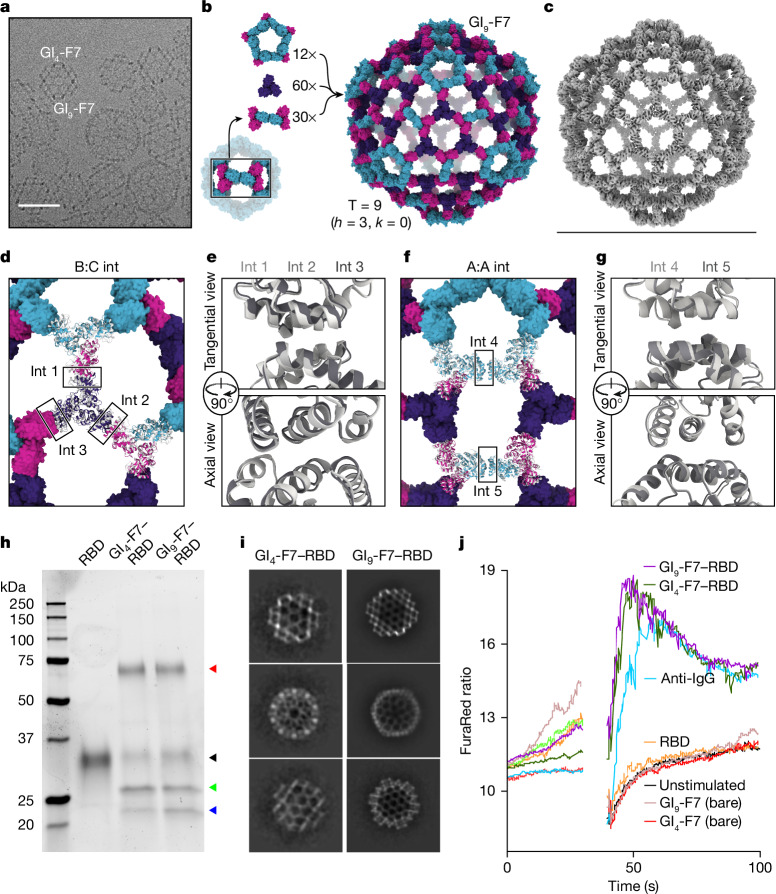
Discovery and characterization of the 540-subunit GI_9_-F7 nanocage. **a**, A cryo-electron micrograph showing GI_4_-F7 and GI_9_-F7 nanocages in the same preparation. Scale bar, 50 nm. **b**, Design model of GI_9_-F7, constructed from 12 pentasymmetrons, 60 CCC homotrimers and 30 disymmetrons. A subunits, cyan; B subunits, magenta; C subunits, purple. **c**, Cryo-EM map of GI_9_-F7 at 6.7 Å resolution. Scale bar, 71 nm. **d**, Comparison of a model derived from the cryo-EM map (grey) with the computational design model (other colours), aligned using a single asu (shown in cartoon). The three independent copies of the B:C interface in the asu are indicated. **e**, Alignment of int 1 (light grey), int 2 (medium grey) and int 3 (dark grey) from the cryo-EM model. **f**, Alignment of two neighbouring copies of AAB heterotrimers from the cryo-EM model to the design model. The two independent copies of the A:A (I3-01) interface, located in the pentasymmetron (int 4) and the disymmetron (int 5), are indicated. **g**, Alignment of the two A:A (I3-01) interfaces from the cryo-EM model, int 4 (light grey) and int 5 (medium grey). **h**, SDS–PAGE of antigen-bearing GI_4_-F7 and GI_9_-F7 nanocages. RBD–SpyTag (left lane) was conjugated to CCC–SpyCatcher in either GI_4_-F7 or GI_9_-F7 nanocages. Red arrowhead, conjugated CCC–RBD; black arrowhead, residual RBD–SpyTag; green arrowhead, A subunit from AAB and ABB and B subunit from BBB; blue arrowhead, B subunit from AAB and ABB. For gel source data, see Supplementary Fig. 5. **i**, Representative 2D class averages from negative-stain electron microscopy of RBD-conjugated GI_4_-F7 or GI_9_-F7 nanocages. **j**, Representative plot of Ca^2+^ flux induced by BCR signalling in RAMOS cells that stably express the SARS-CoV-2 spike-specific antibody COVA2-15 as an IgG BCR^60^. Cell lines were stimulated with various antigens with 4 µg ml^−1^ RBD after reading a 30 s baseline. Data are representative of two independent experiments.

**Extended Data Fig. 5:**
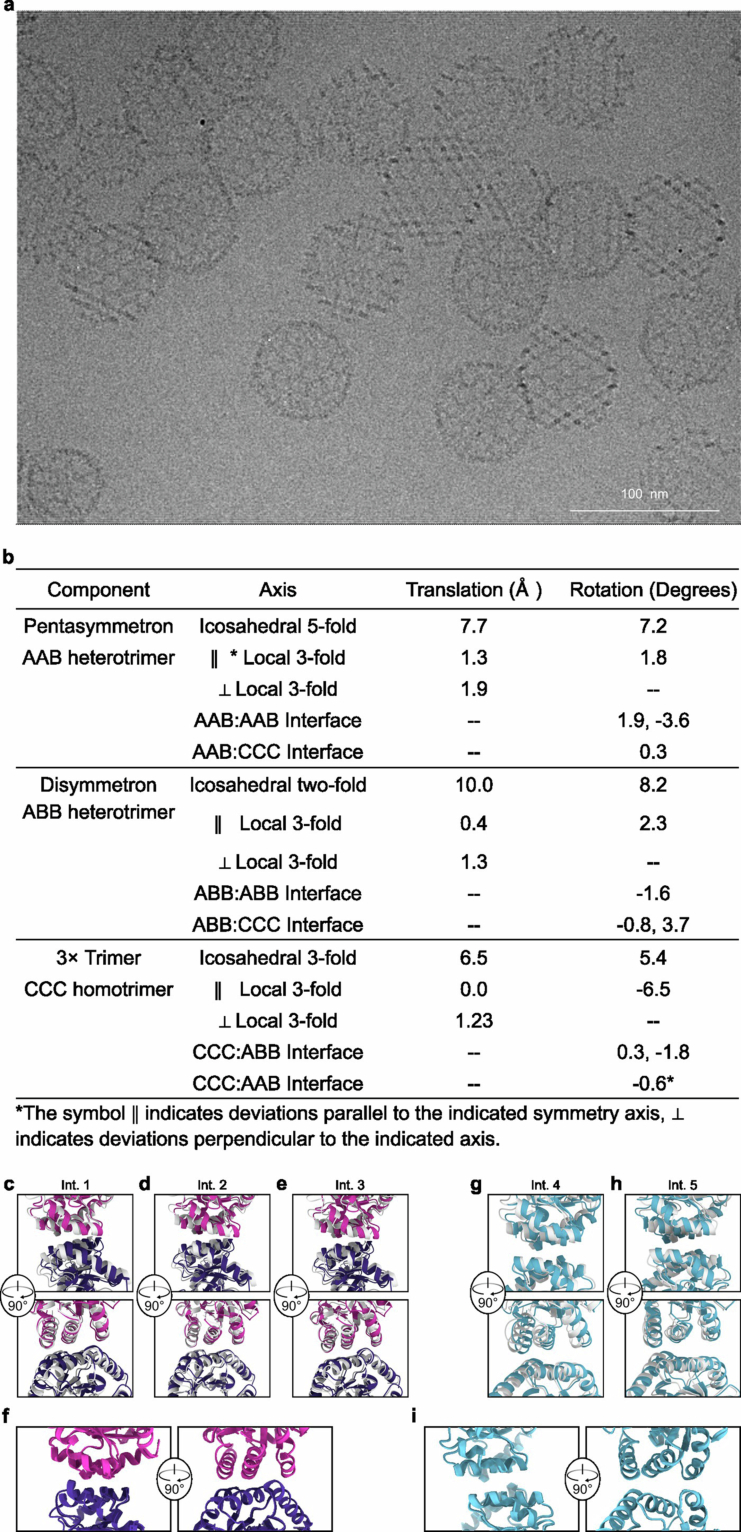
Discovery and structural details of GI_9_-F7. **a**, CryoEM field view micrograph of samples enriched for GI_9_-F7 by SEC purification. Both GI_9_-F7 (large particles) and GI_4_-F7 (e.g., bottom-left corner) are clearly visible. **b**, Table of deviations observed in the cryoEM reconstruction of GI_9_-F7 compared to the design model. (**c-e**) Alignment of the GI_9_-F7 design model chain B (magenta) and chain C (purple) protein-protein interface to the corresponding chains of the cryoEM model (gray). Each of the three interfaces between B and C chains in the asu are shown. **f**, The protein-protein interface between chain B and C from the cryoEM model of GI_4_-F7 (light colors) aligned to the same interface from the cryoEM model of GI_9_-F7 (dark colors). **g**, Alignment of design model to the cryoEM model for the I3-01 interface in the pentasymmetron and g, disymmetron. **h**, Alignment of the I3-01 interface from the cryoEM models of GI_4_-F7 (light blue) and GI_9_-F7 (dark blue).

Unexpectedly, in a number of the GI_4_-F7 micrographs we also observed a 71-nm nanocage with a similar wireframe morphology and hexagonal pores (Fig. 3a). By counting the hexagonal pores we found that *h* = 3; thus the nanocage is T = 9 and we refer to it as GI_9_-F7. GI_9_-F7 can be explained by the presence of small amounts of ABB heterotrimer in AAB heterotrimer preparations. Analogous to the AAB heterotrimer, which forms a pentasymmetron through five roughly two-fold-symmetric A:A interfaces inherited from I3-01, the ABB heterotrimer forms a two-trimer ‘disymmetron’ structure held together by the same A:A interaction (Fig. 3b). In GI_9_-F7 this disymmetron occupies the icosahedral two-fold symmetry axes, providing the edges that connect three-fold-symmetric facets containing three ABB heterotrimers and three CCC homotrimers. As a result, GI_9_-F7 is quasisymmetric in addition to being pseudosymmetric: the A, B and C subunits each occupy multiple, distinct environments in the assembly. We expanded GI_4_-F7 to generate a design model for GI_9_-F7 containing 12 pentasymmetrons constructed from AAB heterotrimers, 30 disymmetrons comprising ABB heterotrimers, and 60 CCC homotrimers (Fig. 3b). The asu of GI_9_-F7 therefore comprises one AAB trimer, one ABB trimer and one CCC trimer. To generate more homogenous preparations of GI_9_-F7, we separately polished the AAB and ABB heterotrimer fractions from IMAC (Methods) and assembled them with CCC homotrimer at a 1:1:1 ratio. Micrographs of SEC-purified GI_9_-F7 assemblies revealed enrichment of the target assembly (Extended Data Fig. 5a), and we determined a cryo-EM structure of the nanocage to 6.7 Å resolution applying icosahedral symmetry, as well as a 4.0 Å resolution structure of the asu (Fig. 3c, Extended Data Fig. 3 and Extended Data Table 2). Consistent with the accuracy with which we designed GI_4_-F7, the GI_9_-F7 cryo-EM structure deviates from the design model by only 11.5 Å Cα r.m.s.d. across all 540 subunits, 1.6% of the nanocage diameter, and superimposition of the designed asu with the structure yields a Cα r.m.s.d. of 4.6 Å across all 9 chains.

Although the pentasymmetron, disymmetron and three homotrimers in GI_9_-F7 are constrained by the five-fold, two-fold and three-fold icosahedral symmetry axes, respectively, no single trimer occupies a position constrained by icosahedral symmetry—the icosahedral three-fold instead passes through a large pore. Each trimer can therefore deviate from the design model along all six rigid-body degrees of freedom. As a result, the two designed nanocage interfaces (B:C and A:A) occupy five quasi-equivalent positions in GI_9_-F7. Two of the B:C interfaces are located within the icosahedral asu, between the CCC homotrimer and the B chain of a neighbouring pentasymmetron (interface 1) or disymmetron (interface 2) (Fig. 3d). The third B:C interface is between the CCC homotrimer and the B chain of a disymmetron in a neighbouring asu (interface 3). Despite being unconstrained by symmetry, interfaces 1 to 3 fit well to the density, with a very small deviation from the design model comprising only a small rotation with very little radial translation (Fig. 3e and Extended Data Fig. 5b–f). Interfaces 4 and 5 are the A:A (that is, I3-01) interfaces in the pentasymmetron and disymmetron, respectively (Fig. 3f). Unlike interfaces 1 to 3, interfaces 4 and 5 appear to differ, with a Cα r.m.s.d. of 1.3 Å to each other and Cα r.m.s.d. values of 2.0 and 2.4 Å to the GI_9_-F7 design model, respectively (Fig. 3g and Extended Data Fig. 5g–i). This difference arises because the pentasymmetron interface (interface 4) is not symmetrically constrained, while the disymmetron interface (interface 5) is constrained by the icosahedral two-fold symmetry axis. We propose that the lowest-energy state of the A:A interface is not perfectly symmetric, but that the symmetry requirements for nanocage assembly force it to adopt a higher-energy, two-fold-symmetric configuration where appropriate.

To gauge the potential utility of GI_4_- and GI_9_-F7 as scaffolds for nanoparticle vaccines, we multivalently displayed the receptor-binding domain (RBD) of the SARS-CoV-2 spike protein on them and measured their ability to activate RBD-specific B cells. We conjugated SpyTagged RBD to GI_4_-F7 and GI_9_-F7 nanocages bearing SpyCatcher^52^ as a genetic fusion on the CCC subunit, yielding a theoretical maximum of 60 and 180 RBDs per nanocage, respectively. Efficient covalent linkage was verified by SDS–PAGE, with excess RBD–SpyTag but no residual CCC–SpyCatcher visible (Fig. 3h). Intact nanocages of the expected size and morphology were observed by negative-stain electron microscopy, although the displayed antigen could not be seen owing to its small size and flexible linkage to each nanocage (Fig. 3i). We then measured Ca^2+^ flux in B cells bearing an RBD-specific B cell receptor (BCR) after treatment with the RBD nanocages, comparing this against monomeric RBD–SpyTag, ‘bare’ nanocages lacking displayed RBD and an anti-IgG positive control that specifically cross-links the transgenic BCRs and provides an estimate of maximal BCR signalling in the assay (Supplementary Fig. 6). The monomeric SpyTag and bare nanocages did not induce BCR signalling above background, whereas the anti-IgG efficiently induced signalling as expected (Fig. 3j). Activation by both RBD-bearing nanocages was more robust than by the anti-IgG control, peaking both faster and higher. These data show that antigen-bearing pseudosymmetric nanocages efficiently activate B cells, suggesting their potential utility as vaccine scaffolds.

## Generation of extensible nanocages

**Fig. 4:**
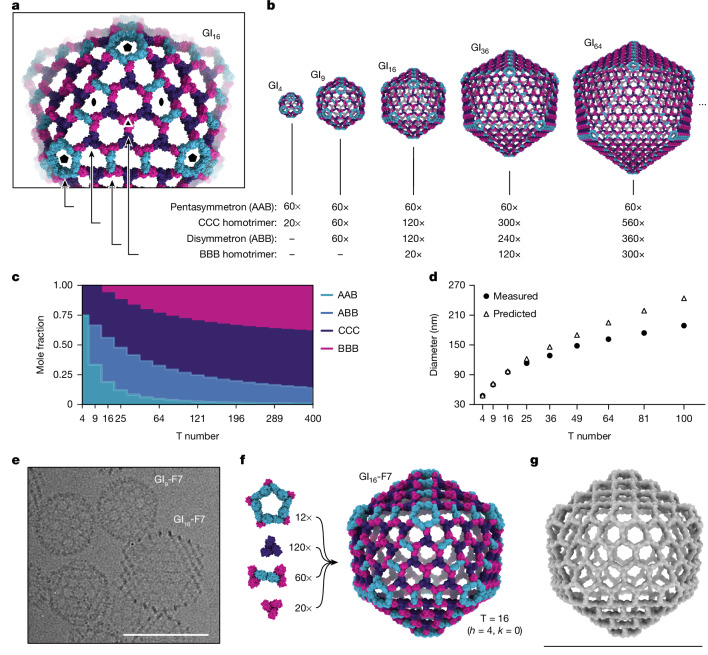
Generation of pseudosymmetric nanocages with extensible hexagonal lattice facets. **a**, The four types of trimers required to generate pseudosymmetric nanocages with T numbers ≥16, viewed in the context of the GI_16_-F7 design model. Icosahedral five-fold, two-fold and three-fold symmetry axes are indicated. **b**, Design models and corresponding building block stoichiometries of GI_4_-F7, GI_9_-F7, GI_16_-F7, GI_36_-F7 and GI_64_-F7 nanocages. The stoichiometries listed indicate the number of each kind of trimeric component. **c**, Graphical depiction of trimer stoichiometry as a function of T number. **d**, Theoretical nanocage diameters and *Z*-average hydrodynamic diameters measured by DLS as a function of trimer stoichiometry used during in vitro assembly (indicated by T number). Data are representative of two independent experiments. **e**, Cryo-electron micrograph of a sample containing GI_9_-F7 and GI_16_-F7 nanocages. Scale bar, 100 nm. **f**, Composition and design model of the GI_16_-F7 nanocage. **g**, The 14.9 Å resolution cryo-EM map of GI_16_-F7. Scale bar, 96 nm.

**Extended Data Fig. 6:**
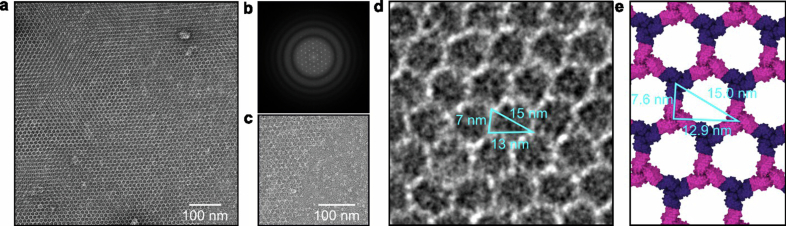
Hexagonal 2D array characterization by negative stain EM. **a**, An example of the regular hexagonal array formed by mixing BBB and CCC homotrimers by negative stain EM. **b**, Power spectrum of the micrograph shown in panel A, confirming the periodic arrangement of the array. **c**, The edge of the array is jagged, with free trimeric components visible. **d**, Measurement of the array dimensions are consistent with **e**, the design model.

The geometries of I3-01 GI_4_-F7, and GI_9_-F7 are analogous to the first three instances in the infinite series of class I Goldberg polyhedra^42,51^. The larger instances in this series are effectively constructed by folding 20 roughly triangular 2D hexagonal lattices into icosahedron-like shapes through the introduction of curvature at their edges and vertices. Theoretically, the next nanocage in the series would be GI_16_-F7. As in GI_4_-F7 and GI_9_-F7, curvature in this structure would be provided by disymmetrons and pentasymmetrons. However, GI_16_-F7 would have a C3-symmetric component centred on the icosahedral three-fold symmetry axis, as opposed to the pore-centred three-fold of GI_9_-F7. Extrapolating from GI_4_-F7 and GI_9_-F7, this component must be a homotrimer of the B chain (‘BBB’), and it must be coplanar with the six surrounding CCC homotrimers (that is, their three-fold axes must be parallel; Fig. 4a). Nanocages beyond GI_16_-F7 simply add more copies of the BBB and CCC homotrimers (and ABB disymmetrons) to form larger two-dimensional hexagonal arrays (Fig. 4b). Thus, obtaining GI_16_-F7 and the larger nanocages in the series does not require new interface design, only production of BBB homotrimer. Indeed, analysing an equimolar mixture of purified BBB and CCC homotrimers by negative-stain electron microscopy yielded a 2D array with a characteristic hexagonal lattice diffraction pattern (Extended Data Fig. 6a–c). The dimensions of the array agree well with a design model derived from the GI_9_-F7 nanocage (Extended Data Fig. 6d,e).

In the absence of other control mechanisms, the inclusion of BBB homotrimer in assembly reactions should yield distributions of large T number assemblies rather than monodisperse preparations of a single species. However, the relative stoichiometries of the components in each assembly vary as a function of T number (Fig. 4b,c), providing a potential mechanism for modulating assembly size. We prepared assembly reactions containing the 4 components at the stoichiometries corresponding to T = 4, 9, 16, 25, 36, 49, 64, 81 and 100 nanocages. Consistent with our predictions, the *Z*-average hydrodynamic diameter measured by dynamic light scattering (DLS) increased with increasing target T number (from 47.5 ± 0.4 nm to 188 ± 1.1 nm), though the observed hydrodynamic diameter deviated from the predicted diameter at higher T numbers (Fig. 4d and Extended Data Table 3). This deviation could be due to contaminating AAB trimer in the ABB fraction (Extended Data Fig. 2d), which would be expected to favour the lower T number assemblies in which AAB is more prevalent. Furthermore, smaller assemblies will be kinetically favoured over larger assemblies, which could bias the resulting particle distribution. GI_16_-F7 was readily observed by cryo-EM in assembly reactions prepared at the T = 16 stoichiometry (Fig. 4e). GI_16_-F7 is predicted to have a diameter of 96 nm and contains 12 pentasymmetrons, 120 CCC homotrimers, 60 disymmetrons and 20 BBB homotrimers for a total of 960 subunits (Fig. 4f). We determined a 14.9 Å resolution cryo-EM map of GI_16_-F7 and found that it closely matches the expected geometry of the design (Fig. 4g). This assembly has an internal volume that is roughly 90-fold larger than our previously designed nanocages with strict icosahedral symmetry^11,14^ and adeno-associated viruses, commonly used vectors for gene therapy^53^.

## Conclusions

Here we show that designing pseudosymmetric protein building blocks, in which symmetry is broken at the sequence level while backbone symmetry is maintained, enables the construction of very large pseudosymmetric protein nanocages. This work moves beyond established methods for accurately designing novel self-assembling proteins^10,11,14,54^, as it breaks their reliance on strict symmetry and provides a route to a large set of architectures that were previously inaccessible to design. Although both the previous and current methods are general with respect to the choice of building block and can therefore give rise to rich varieties of potential assemblies, the space of asymmetric architectures is vastly larger than that of strictly symmetric structures.

For this work, we used a hyperstable protein from a thermophilic organism as a building block, as many studies have shown that stable proteins are more tolerant of modification^55^. Although this choice contributed to the successful expression of a large number of mutants, it also led to the low success rate of symmetry-breaking mutations: 1WA3 proved remarkably resilient to mutations intended to disrupt the homotrimer. Key to our success was having an efficient screen for connecting genotype to phenotype (in this case, maintenance of backbone symmetry), which we achieved by selecting a building block that had already been used as a component in a larger symmetric assembly. Although at present this approach may limit the use of our experimental screen to a subset of known protein oligomers, our overall design strategy in theory generalizes to any oligomeric protein. We expect this limitation to further diminish as methods for protein structure prediction and design continue to yield improved success rates, which will enable the generation of increasingly asymmetric protein nanomaterials (see the accompanying Article^56^).

Although some small viruses make purely pseudosymmetric capsids, many larger capsids are constructed by combining pseudosymmetry with quasisymmetry. Analogously, whereas GI_4_-F7 and the assemblies reported in the accompanying manuscript^56^ are pseudosymmetric, with each distinct subunit in a single chemical environment, GI_9_-F7 and its larger counterparts are also quasisymmetric, with genetically identical subunits in more than one chemical environment. The A subunit occupies an asymmetric position in the pentasymmetron and either an asymmetric position in the disymmetron (for even T numbers) or both asymmetric and two-fold symmetric positions in disymmetrons (for odd T numbers greater than nine). Similarly, the B and C subunits occupy different chemical environments depending on their locations in the assembly. Quasisymmetry is enabled by the use of two-component heterotrimers (ABB and AAB), which provides for economy in coding for larger assemblies. The T = 4 structure requires only 3 distinct chains, compared with 4 chains for the more conceptually straightforward approach of a strictly pseudosymmetric ABC heterotrimer and DDD homotrimer. For larger particles the economy is even greater: for example, only 3 unique chains are required to make T = 9 nanocages, but 7 would be needed for the strictly pseudosymmetric approach. The trade-off to this economy is a reduction in precision compared with the approach described in the accompanying Article^56^, although as we have shown, this can be partially overcome by modulating the stoichiometry of the assembly reaction.

We used a hierarchical design strategy to fulfil the requirement for multiple designed interfaces in our pseudosymmetric nanomaterials. After first constructing pseudosymmetric heterotrimers and combining these with an existing designed interface to generate pentasymmetrons, producing 240-subunit and larger pseudosymmetric assemblies required only one additional dock-and-design step. Similar hierarchical and modular design strategies are widespread in reticular chemistry^57^ and DNA nanotechnology^58^, and should become increasingly powerful in protein nanomaterials design as the number and kinds of modular protein building blocks continue to increase^59^.

## Methods

### Pseudosymmetric trimer design

To identify mutations for altering trimer assembly specificity, we first identified all pairs of interacting residues in the trimer interface. Contacts were defined as any residue with a heavy (that is, non-hydrogen) atom within 4 Å of a heavy atom in a residue across the interface. We then used Rosetta to calculate the total score of poses containing all possible pairs of mutations, as well as the difference in score between the trimeric and monomeric states using the ddG filter. Example scripts are provided as supplementary files. Individual mutations were evaluated by comparing their ddG and total scores to those of the wild-type (WT) interface according to equation (1). The total scores and ddG values of the paired mutations were similarly normalized according to equation (2).

1 $$\begin{array}{l}{\rm{Single}}\,{\rm{mutant}}\,\Delta {\rm{score}}={{\rm{score}}}_{{\rm{mutant}}}-{{\rm{score}}}_{{\rm{WT}}};\\ {\rm{Single}}\,{\rm{mutant}}\,\Delta {\rm{ddG}}={{\rm{ddG}}}_{{\rm{mutant}}}-{{\rm{ddG}}}_{{\rm{WT}}}\end{array}$$

2 $$\begin{array}{l}{\rm{Double}}\,{\rm{mutant}}\,\Delta {\rm{score}}={{\rm{score}}}_{{\rm{mutant}}}-{{\rm{score}}}_{{\rm{WT}}};\\ {\rm{Double}}\,{\rm{mutant}}\,\Delta {\rm{ddG}}={{\rm{ddG}}}_{{\rm{mutant}}}-{{\rm{ddG}}}_{{\rm{WT}}}\end{array}$$

Ideal mutant pairs were those where one or both single mutations increased the energy of the trimer relative to the wild type (that is, normalized scores > 0) while the double mutation had no effect or stabilized the trimer (that is, normalized scores ≤ 0). We also identified likely positions for design using coevolutionary analysis^48,49^. Strongly co-evolving residues at the protein–protein interface were identified using GREMLIN. We then identified mutations that were negatively correlated with the wild-type pair for testing experimentally.

### Mutant protein expression

Mutant I53-50A trimers were expressed at three scales. Small-scale expression was performed at 1 ml culture volume in 96-well plates with 2 ml well volume. Medium-scale expressions were performed at 50 ml culture volume in 250 ml baffled shake flasks. Large-scale expressions were performed at 500 ml culture volumes in 2 l baffled shake flasks. All proteins were expressed in T7 competent *E. coli* in TB medium, with IPTG induction for 3 h at 37 °C. Cells were pelleted and frozen at −20 °C until lysis. Prior to lysis cells were defrosted on ice in lysis buffer (50 mM Tris pH 8.0, 250 mM NaCl, 20 mM imidazole, 1 mM phenylmethylsulfonyl fluoride, 1 mM dithiothreitol (DTT), 0.1 mg ml^−1^ DNase, and 0.1 μM RNase, unless otherwise noted). Small-scale expressions were lysed with a plate sonicator (QSonica), medium-scale expressions were lysed with a probe sonicator, and large-scale expressions were lysed by microfluidization (18,000 psi, one pass). Lysates from small-scale expressions were clarified by centrifugation in a swinging bucket rotor at 4,000*g*. Lysates from medium- and large-scale expression lysates were clarified by centrifugation at 12,000*g* in a fixed-angle rotor.

### I53-50B expression and purification

Pentameric I53-50B was produced recombinantly in *E. coli*. A pET29b expression plasmid encoding I53-50B.4PT1^11^ was synthesized by GenScript using the NdeI and XhoI restriction sites with a double stop codon just before the C-terminal polyhistidine tag. Tagless protein was expressed in Lemo21(DE3) cells (NEB) in LB (10 g Tryptone, 5 g Yeast Extract, 10 g NaCl) grown in a 10 l BioFlo 320 Fermenter (Eppendorf). At inoculation, impeller speed was set to 225 rpm, gas flow rate was set to 5 standard litres per minute with O_2_ supplementation as part of the dissolved-oxygen aeration cascade, and the temperature set to 37 °C. At the onset of a dissolved oxygen spike (OD ~ 12), the culture was fed with a bolus addition of 100 ml of 100% glycerol and induced with 1 mM IPTG. During this time, the culture temperature was reduced to 18 °C and O_2_ supplementation was ceased, with expression continuing until OD reached ~20. The culture was collected by centrifugation and the protein was purified from inclusion bodies. First, pellets were resuspended in PBS, homogenized, and then lysed by microfluidization using a Microfluidics M110P at 18,000 psi. Following sample clarification by centrifugation (24,000*g* for 30 min), the supernatant was discarded and protein was extracted from the pellet using a series of three washes. The first wash consisted of PBS, 0.1% Triton X-100, pH 8.0. The second wash consisted of PBS, 1 M NaCl, pH 8.0, and the final wash (extraction) consisted of PBS, 2 M urea, 0.75% CHAPS (3-[(3-cholamidopropyl)dimethylammonio]-1-propanesulfonate), pH 8.0. Following extraction, the sample was applied to a DEAE Sepharose FF column (Cytiva) on an AKTA Avant150 FPLC system (Cytiva). After sample binding, the column was washed with 5 column volumes of PBS at pH 8.0 with 0.1% Triton X-100, followed by a wash with 5 column volumes of PBS at pH 8.0 with 0.75% CHAPS. The protein was eluted with 3 column volumes of PBS at pH 8.0 with 500 mM NaCl. After purification, fractions were pooled and concentrated in 10K MWCO centrifugal filters (Millipore), sterile filtered (0.22 μm), aliquoted and flash-frozen in liquid nitrogen, and stored at −80 °C until use.

### Assembly competency analysis

Single mutations were introduced into the I53-50A trimer^11^ by QuikChange site-directed mutagenesis. Sequence-verified mutants were expressed at small scale. Clarified lysates were separated from pellets and a 5 µl aliquot was set aside for characterization by SDS–PAGE. The pellet was resuspended in lysis buffer and a 5 µl aliquot was set aside for characterization by SDS–PAGE. Clarified lysate was immediately mixed with purified I53-50B.4PT1 pentamer. Because trimer expression levels varied from mutant to mutant, pentamer was added at three different concentrations. To 10 µl lysate, 7.5, 2.5 or 0 µl lysis buffer was added, followed by 2.5, 7.5 or 10 µl I53-50B.4PT1 pentamer at 1.8 mg ml^−1^. The assembly reaction was allowed to proceed for 30 min at room temperature. Purified I53-50B.4PT1 pentamer was included on all native PAGE gels. A 10 µl aliquot of each assembly reaction was mixed 1:1 with Native Sample Buffer (Bio-Rad Laboratories), loaded into precast 4–15% polyacrylamide gels (Bio-Rad Laboratories), and run with 1× Tris-Glycine Native PAGE buffer for 3 h at 200 V. The gel was stained with GelCode Blue (Thermo Fisher Scientific) and destained in water. The lack of an I53-50 nanocage band on the native gel indicated single mutations that disrupted either trimer formation or trimer geometry such that the mutant trimer was no longer assembly-competent.

### Screening of mutant combinations

Single mutants that disrupted I53-50A trimer—and therefore I53-50 nanocage—formation were combined with ‘rescue’ mutations intended to generate pseudosymmetric I53-50A trimers. Synthetic DNA encoding potential combinations were ordered as heterotrimeric operons cloned into pCDB179 from IDT. To facilitate detection of the distinct components of the heterotrimer, a 6×His-SUMO domain was added to one subunit and sfGFP and an avi-tag added to a second subunit via genetic fusion. The third subunit bore a Strep-tag via genetic fusion. Variants were tested for I53-50 nanocage formation using trimer-containing *E. coli* lysates and purified I53-50B pentamer as described above. Combinations that formed I53-50 nanocages were expressed at large scale and purified by Ni^2+^ affinity chromatography on a HisTrap FF column (Cytiva). In brief, clarified lysate was passed through a pre-equilibrated 5 ml HisTrap FF column, washed with 3–5 column volumes of wash buffer (50 mM Tris pH 8.0, 250 mM NaCl, 20 mM imidazole, 1 mM DTT), and heterotrimer was eluted with either a step elution or a gradient over 40 min at 3 ml min^−1^ flow rate into 100% elution buffer (50 mM Tris pH 8.0, 250 mM NaCl, 500 mM imidazole, 1 mM DTT). Major fractions corresponding to the two observed peaks in the elution profile were pooled separately, concentrated in a 30-kDa cut-off Amicon concentrator (Millipore), and injected onto a pre-equilibrated Superdex 200 Increase 10/300 column (Cytiva). The SEC buffer was 25 mM Tris pH 8.0, 150 mM NaCl, 1 mM DTT. Fractions corresponding to the trimer peak from each chromatogram were collected for analysis by native mass spectrometry. Alternatively, the IMAC eluate was pooled and loaded onto a StrepTrap HP column (Cytiva) pre-equilibrated in binding buffer (100 mM Tris pH 8.0, 150 mM NaCl, 1 mM EDTA, and 1 mM DTT). The column was then washed with 10 column volumes of binding buffer, or until the A280 absorbance leveled off at baseline and eluted with a step elution in binding buffer plus 2.5 mM desthiobiotin. Major fractions were analysed by reducing SDS–PAGE.

### Native mass spectrometry

Trimer purity, identity, and oligomeric state were analysed by on-line buffer-exchange mass spectrometry^61^ in 200 mM ammonium acetate using a Vanish ultra-performance liquid chromatography coupled to a Q Exactive ultra-high mass range Orbitrap mass spectrometer (Thermo Fisher Scientific). The recorded mass spectra were deconvolved with UniDec version 4.2+ (ref. ^62^).

### Assembly of I53-50 nanocages using pseudosymmetric I53-50A heterotrimers

The native mass spectrometry-verified pseudosymmetric I53-50A heterotrimer was expressed and purified at medium scale as described above and mixed at a 1:1 molar ratio with purified I53-50B.4PT1 pentamer and allowed to assemble at room temperature for 30 min. Assembled nanocages were characterized by DLS and negative-stain electron microscopy as described below.

### Computational design of T = 4 nanocages

We created a model of the pentasymmetron by extracting five trimers surrounding the icosahedral five-fold from I3-01^14^. We reverted the interface residues on the unpaired subunit back to the original 1WA3 sequence, mutated 12 residues to negatively charged amino acids to enhance expression and facilitate purification, then combined each trimer into a single chain so that the pentasymmetron could be treated computationally as a simple homopentamer. We used previously described protocols^11^ to dock and design T = 4 nanocages, with some modifications to the design script. Example design scripts are provided as supplementary files. Docked configurations were manually screened to ensure interfaces were between the unpaired pentasymmetron subunit and the homotrimer. Designs were visually inspected and any overly exposed hydrophobic residues introduced during design were reverted to their wild-type identities.

### Screening of T = 4 nanocages by co-purification

Three tricistronic genes were ordered from IDT. An N-terminal GFP was included on the A subunit of the pentasymmetron heterotrimer as a mass tag. A C-terminal 6×His tag was added to the C subunit. Genes were expressed at medium scale. Clarified lysate was loaded onto 1 ml of Ni-NTA resin (Thermo Fisher Scientific) pre-equilibrated in wash buffer. After washing with three column volumes of wash buffer, the protein was eluted with two column volumes of elution buffer. Eluate was screened for the presence of all three gene products by SDS–PAGE.

### Purification of co-expressed GI_4_-F7

GI_4_-F7 nanocages expressed tricistronically at large scale were purified by loading on a 5 ml HisTrap FF column (Cytiva) equilibrated in wash buffer (50 mM Tris pH 8.0, 250 mM NaCl, 20 mM imidazole, 1 mM DTT). After loading, the column was washed with 3–5 column volumes of wash buffer and protein was eluted with a gradient into 100% elution buffer (50 mM Tris pH 8.0, 250 mM NaCl, 500 mM imidazole, 1 mM DTT) over 40 min at 3 ml min^−1^. The major fractions from elution were pooled, concentrated to ~1 ml, and loaded onto an equilibrated Sephacryl S-500 HR 10/300 GL. SEC buffer was 25 mM Tris pH 8.0, 150 mM NaCl, 1 mM DTT.

### Purification of GI_4_-F7 heterotrimeric and homotrimeric components

For in vitro assembly, the heterotrimeric component of GI_4_-F7, comprising only the A and B chains, was expressed bicistronically. The A chain was modified with an N-terminal 6×His tag. When expressed this way, some AAA nanocages and BBB homotrimers probably assemble in addition to AAB and ABB heterotrimers. To purify AAB from ABB heterotrimers, the bicistronic gene was expressed at large scale with the modification that 0.75% CHAPS was added to the lysis buffer and DTT was omitted. Clarified lysate was purified with a 5 ml HisTrap FF column as described above. Elution chromatograms contained three peaks. The first peak was predominantly the ABB heterotrimer, the second peak was predominantly AAB heterotrimer, and the third peak was predominantly AAA homotrimers assembled into an I3-01-like particle. Any BBB homotrimer would be in the flow-through. The first and second peaks were pooled separately and concentrated to ~1 ml. To remove any residual I3-01-like nanocage, we further purified the concentrated fractions on a Superose 6 Increase 10/300 column. The SEC buffer was 25 mM Tris pH 8.0, 150 mM NaCl, 0.75% w/v CHAPS. Glycerol was added to purified heterotrimer to a final concentration of 5%, the concentration was determined by A280, and 1 ml aliquots were flash-frozen in liquid nitrogen. Aliquots were stored at −80 °C until use. The homotrimer components were expressed at large scale and purified by IMAC in the same way as the co-expressed GI_4_-F7 nanocages except that 1% CHAPS was added to all buffers. It was further purified by SEC on a HiLoad 26/600 Superdex 200 PG column in 25 mM Tris pH 8.0, 150 mM NaCl, 5% glycerol, 1.0% w/v CHAPS, 1 mM DTT. The total trimer protein concentration was measured by A280, flash-frozen in liquid nitrogen in 1 ml aliquots, and stored at −80 °C until use.

### In vitro assembly of GI_T_-F7 nanocages

To assemble GI_T_-F7 nanocages, components were mixed at various stoichiometries depending on the target assembly state in the presence of 3% CHAPS, a condition that prevented premature assembly. This was necessary to prevent the assembly of off-target species during addition of the multiple components required to generate the target assemblies. For example, mixing BBB–CCC heterotrimers prior to the addition of AAB and ABB components under assembly-permissive conditions would result in 2D arrays instead of GI_T_-F7 nanocages (see Extended Data Fig. 6). Once all components were added, the mixtures were dialysed into 0% CHAPS overnight at room temperature in a 30-kDa cut-off dialysis cassette. As an extra precaution, the AAB and ABB heterotrimers were mixed first since they do not directly interact with the BBB homotrimer, followed by addition of the CCC homotrimer. Nanocages were prepared fresh for each experiment, or stored at 4 °C for up to three days. To assemble BBB–CCC 2D arrays, the components were first individually dialysed to remove CHAPS, and then mixed at a 1:1 stoichiometric ratio and allowed to assemble overnight at room temperature.

### Characterization of assemblies

Assemblies were characterized in solution by DLS. Samples were measured in triplicate, technical replicates, using an UNcle (UNchained Labs) according to the manufacturer’s directions. In brief, 8.8 µl of sample was loaded in triplicate into the capillary cassette. For each replicate, 10 acquisitions 10 s in length were collected. Assemblies were further characterized by negative-stain electron microscopy. Samples were diluted to between 0.1 and 0.5 mg ml^−1^ total protein depending on the assembly stoichiometry, applied to a glow-discharged thick carbon film 400 mesh copper grid (Electron Microscopy Sciences), and stained with 2% uranyl formate. Care was taken to ensure the stain thickness was sufficient to support the larger assemblies. Micrographs were collected on a Talos L120C (FEI) at up to 48,000× magnification. Individual micrographs were processed with ImageJ.

### Conjugation of RBD antigens to GI_T_-F7 nanocages and characterization by negative-stain electron microscopy

To enable conjugation of antigens to assembled nanocages, CCC trimers were fused to a SpyCatcher002 motif at their C terminus, expressed in *E. coli*, and purified via IMAC and SEC, as described above. Nanocages were then assembled at the appropriate stoichiometries for T = 4 and T = 9 assemblies by dialysing into a 0% CHAPS solution overnight. Assembled particles were then mixed with an excess of RBD-SpyTag002 and mixed at 4 °C for 3 h. Conjugation was confirmed by SDS–PAGE, wherein the mass of RBD showed a ~30 kDa increase, consistent with conjugation to CCC proteins in nanocage assemblies. After conjugation, particles were also visualized by negative-stain electron microscopy to confirm intact assemblies. Samples were prepared by applying 3 µl of a 5 µM nanocage solution to glow-discharge carbon-coated grids, followed by staining with uranyl formate 3 times prior to imaging. EPU software (Thermo Fisher) was used to collect at least 100 micrographs of each sample. Images were imported to CryoSparc and particles were averaged to obtain initial 2D classes. Selected classes were then used to generate templates for a second round of particle picking, and new particles were averaged multiple times to obtain the 2D classes shown in Fig. 3i.

### B cell activation assay

The COVA2-15 IgG RAMOS cell line was generously provided by the van Gils laboratory^60^ and not authenticated further. For Ca^2+^ flux experiments, cells were loaded with FuraRed cell-permeable dye (Thermo Fisher) for 30 min in RPMI1640 supplemented with 10% fetal clone II, 1% l-glutamax, and 1% penicillin–streptomycin (complete medium) at a cell concentration of 1 × 10^7^ per ml. Cells were then washed with 10× volume complete medium, resuspended at 2 × 10^6^ cells per ml in complete medium, and aliquoted at 0.25 ml into individual FACS tubes. Samples were kept at room temperature and then warmed in a 37 °C bath for 3 min immediately before use. Acquisition was performed on an Attune CytPix flow cytometer (Thermo Fisher) with baselines recorded for 30 s for each sample before addition of antigen and measurement of BCR-specific activation. For gating strategy, see Supplementary Fig. 6. A polyclonal goat anti-human IgG F(ab′)2 (Southern Biotech) was used as a positive control for signalling resulting from IgG BCR cross-linking by addition of 2.5 µg to cells. The FuraRed ratio of bound (fluorescence in VL3) and unbound (fluorescence in BL1) Ca^2+^ was used for analysis using FlowJo v10 (BD Biosciences). Cell lines were not tested for mycoplasma.

### Cryo-EM sample preparation, data collection and data processing

Three microlitres of 3 mg ml^−1^ GI_4_-F7, GI_9_-F7, and GI_16_-F16 were loaded onto freshly glow-discharged R 2/2 UltrAuFoil grids, prior to plunge freezing using a Vitrobot Mark IV (Thermo Fisher Scientific) with a blot force of 0 and 6 sec blot time at 100% humidity and 22 °C. Data were acquired using an FEI Titan Krios transmission electron microscope operated at 300 kV and equipped with a Gatan K3 direct detector and Gatan Quantum GIF energy filter, operated in zero-loss mode with a slit width of 20 eV. For GI_4_-F7 and GI_9_-F7, automated data collection was carried out using Leginon^63^ at a nominal magnification of 105,000× with a pixel size of 0.843 Å. 7,249 and 2,558 micrographs were collected with a defocus range comprised between −0.5 and −2.5 μm, respectively. The dose rate was adjusted to 15 counts per pixel per s, and each movie was acquired in super-resolution mode fractionated in 75 frames of 40 ms. For the GI_16_-F7 data set, automated data collection was carried out using Leginon^63^ at a nominal magnification of 64,000× with a pixel size of 1.42 Å. In total, 2,268 micrographs were collected with a defocus range between −0.5 and −3.5 μm. The dose rate was adjusted to 15 counts per pixel per s, and each movie was acquired in super-resolution mode fractionated in 50 frames of 100 ms. Movie frame alignment, estimation of the microscope contrast-transfer function parameters, particle picking and extraction were carried out using Warp^64^.

Two rounds of reference-free 2D classification were performed using CryoSPARC^65^ to select well-defined particle images. These selected particles were subjected to two rounds of 3D classification with 50 iterations each (angular sampling 7.5° for 25 iterations and 1.8° with local search for 25 iterations) using Relion^66^ with an initial model generated with ab initio reconstruction in cryoSPARC. 3D refinements were carried out using non-uniform refinement along with per-particle defocus refinement in CryoSPARC. Selected particle images were subjected to the Bayesian polishing procedure^67^ implemented in Relion 3.1 before performing another round of non-uniform refinement in cryoSPARC followed by per-particle defocus refinement and again non-uniform refinement. To further improve the density of the asu, the particles were symmetry-expanded and subjected to focus 3D classification without refining angles and shifts. Particles belonging to classes with the best resolved asu density were selected and then subjected to local refinement using CryoSPARC. Local resolution estimation,and sharpening were carried out using CryoSPARC. Reported resolutions are based on the gold-standard Fourier shell correlation of 0.143 criterion and Fourier shell correlation curves were corrected for the effects of soft masking by high-resolution noise substitution^68,69^.

### Model building and refinement

UCSF Chimera^70^ and Coot^71^ were used to fit atomic models into the cryo-EM maps. GI_4_-F7 and GI_9_-F7 asu models were refined and relaxed using Rosetta using sharpened and unsharpened maps^72,73^. For GI_4_-F7 or GI_9_-F7 icosahedral model, all of the side chains of GI_4_-F7 or GI_9_-F7 asu model are truncated except Gly, Cys, and Pro residues and the symmetry-related copies were generated in ChimeraX with cryo-EM maps.

### Alignments and images

To align the cryo-EM models to the design model, both models were centred at the origin and their icosahedral symmetry axes aligned in PyMOL^74^. The Cα r.m.s.d. was calculated using the rms_cur function in PyMOL. To measure deviations in the rigid-body degrees of freedom, copies of the pentasymmetron, disymmetron, and trimer (or trimers for GI_9_-F7) from the cryo-EM model were aligned to the design model using the ‘super’ function in PyMOL. We then calculated the rotations and translations from the transformation matrix between the corresponding component of the original cryo-EM model and the aligned cryo-EM model. We applied the same approach to the heterotrimer (and homotrimer for GI_9_-F7) components to obtain rotations and translations within the pentasymmetron, disymmetron, and homotrimer components, respectively. We found that the ‘super’ function in PyMOL was very sensitive to chain and residue numbering, as well as some of the minor differences between the design model and cryo-EM model. Therefore, for all alignments using PyMOL, we made sure to harmonize residue numbering, chain IDs, and remove any residues present in only one model or the other. For that reason, aligned images were generated using the mm command in ChimeraX^75^ and verified to ensure that the alignments closely matched those generated on the trimmed models created with the super function in PyMOL.

### Scripts and plots

All data were processed and plotted using Python 3.8.8, matplotlib 3.3.4 and seaborn 0.11.1.

### Reporting summary

Further information on research design is available in the Nature Portfolio Reporting Summary linked to this article.

## Online content

Any methods, additional references, Nature Portfolio reporting summaries, source data, extended data, supplementary information, acknowledgements, peer review information; details of author contributions and competing interests; and statements of data and code availability are available at 10.1038/s41586-024-08360-6.

## Supplementary information

Supplementary Information **Gel** images, flow cytometry gating strategy and table of all novel sequences. Five supplementary gel images provide uncropped SDS–PAGE images of representative single-mutant assemblies, representative double-mutant assemblies, IMAC and StrepTrap purification, nanocage screening, and antigen-nanocage conjugation. The flow cytometry gating strategy used for B cell activation assays is also provided, as well as a table listing all novel amino acid sequences used in this study.

Reporting Summary

## Figures and Tables

**Extended Data Table 1:**
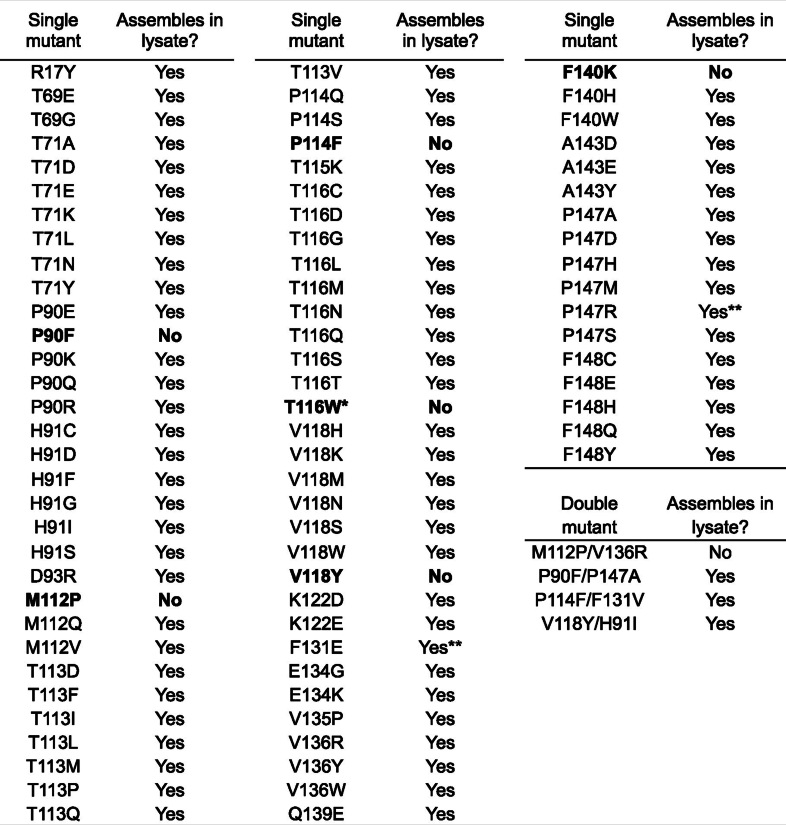
Table of all single and double mutants and outcome

* Mutant at three-fold position required screening in a tricistronic gene construct and was not pursued further.

** Did not assemble in one replicate or had a weak assembly band.

**Extended Data Table 2:**
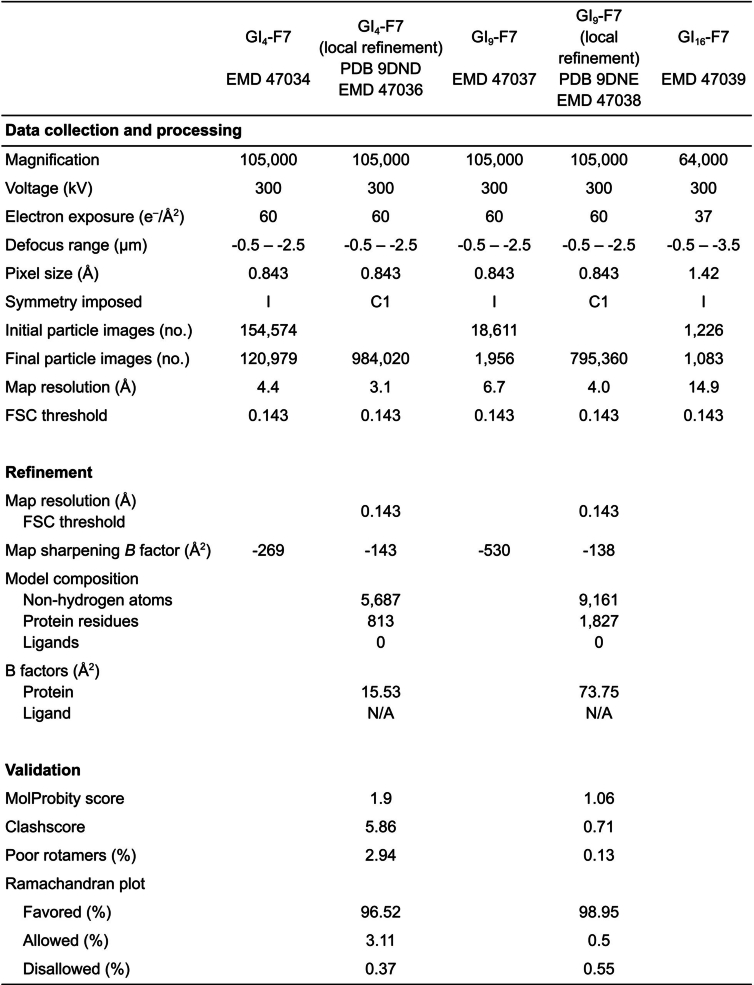
CryoEM data collection and refinement statistics

**Extended Data Table 3:**
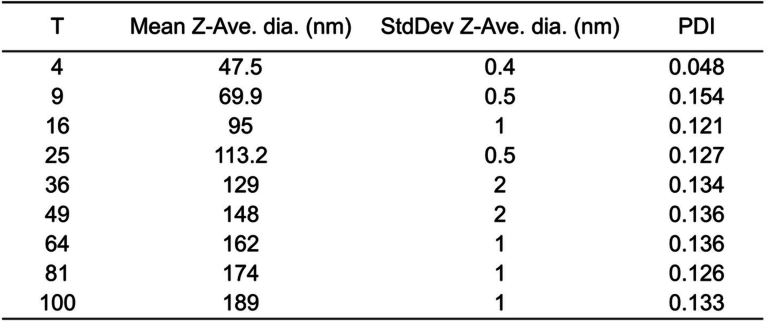
DLS results obtained from assembly reactions corresponding to T numbers 4 to 100

## Data Availability

Electron microscopy maps and models for GI_4_-F7 are available from the Electron Microscopy Data Bank (EMD) under accession number EMD-47034, local refinements for GI_4_-F7 are available under accession number EMD-47036 and also in the Protein Data Bank (PDB) under accession number 9DND, electron microscopy maps and models for GI_9_-F7 are available under accession number EMD-47037, local refinements for GI_9_-F7 are available under PDB ID 9DNE and EMD-47038, and electron microscopy maps and models for GI_16_-F7 are available under accession number EMD-47039. Structural data for the KDPG from *T. maritima*, the lumazine synthase from *Mesorhizobium loti* and I3-01 are available in the Protein Data Bank (PDB IDs 1WA3, 2OBX and 8ED3, respectively). All other data are available in the manuscript or the supplementary materials.

## Code availability

Example.xml scripts, command lines, and a README file are available on GitHub at https://github.com/quecloud/Hierarchical-pseudosymmetric-nanocage-design and through Zenodo at 10.5281/zenodo.13958626 (ref. ^76^).
