# Supplementary material for: Hierarchical design of pseudosymmetric protein nanocages
Source: Nature. 2024 Dec 18;638(8050):553–61. doi: 10.1038/s41586-024-08360-6 (PMC11821544; doi:10.1038/s41586-024-08360-6)
Supplement: Supplementary file 1 — Gel images, flow cytometry gating strategy and table of all novel sequences. Five supplementary gel images provide uncropped SDS–PAGE images of representative single-mutant assemblies, representative double-mutant assemblies, IMAC and StrepTrap purification, nanocage screening, and antigen-nanocage conjugation. The flow cytometry gating strategy used for B cell activation assays is also provided, as well as a table listing all novel amino acid sequences used in this study. [file 41586_2024_8360_MOESM1_ESM.pdf]

nature portfolio

https://doi.org/10.1038/s41586-024-08360-6

# Supplementary information

# Hierarchical design of pseudosymmetric protein nanocages

In the format provided by the
authors and unedited

# Supplementary Information

# Hierarchical design of pseudosymmetric protein nanocages

Quinton M. Dowling[1,2], Young-Jun Park[3], Chelsea N. Fries[2,3], Neil Gerstenmaier[2,3], Sebastian Ols[2,3], Erin C. Yang[2,3], Adam Wargacki[2,3], Annie Dosey[2,3], Yang Hsia[2,3], Rashmi Ravichandran[2,3], Carl Walkey[2,3], Anika Burrell[3], David Veesler[3,4], David Baker[2,3,4], Neil P. King[2,3]

[1]Department of Bioengineering, University of Washington, Seattle, WA 98195, USA
[2]Institute for Protein Design, University of Washington, Seattle, WA 98195, USA
[3]Department of Biochemistry, University of Washington, Seattle, WA 98195, USA
[4]Howard Hughes Medical Institute, Seattle, WA 98195, USA

**Table of Contents**

**Supplementary Fig. 1:** Full SDS-PAGE image of representative single-mutant assemblies 3
**Supplementary Fig. 2:** Full SDS-PAGE image of representative double-mutant assemblies 4
**Supplementary Fig. 3:** Full SDS-PAGE image IMAC and StrepTrap purification gel 5
**Supplementary Fig. 4:** Full SDS-PAGE image of nanocage screening gel 6
**Supplementary Fig. 5:** Full SDS-PAGE image of antigen-nanocage conjugation 7
**Supplementary Fig. 6:** B cell activation study gating strategy 8
**Supplementary Table. 1:** List of all novel amino acid sequences used in this study 9

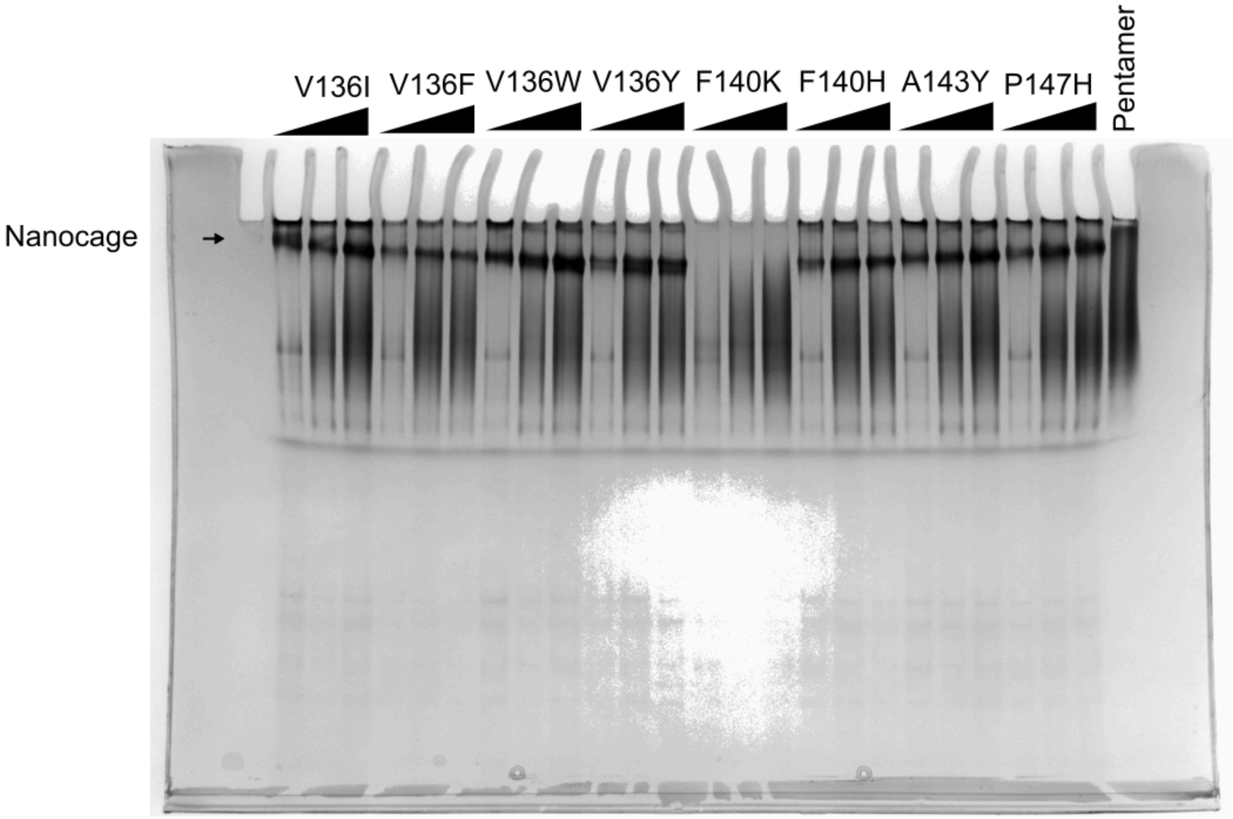


**Supplementary Fig. 1:** Uncropped Native-PAGE image of representative single-mutant assemblies from Main Text Fig. 1.

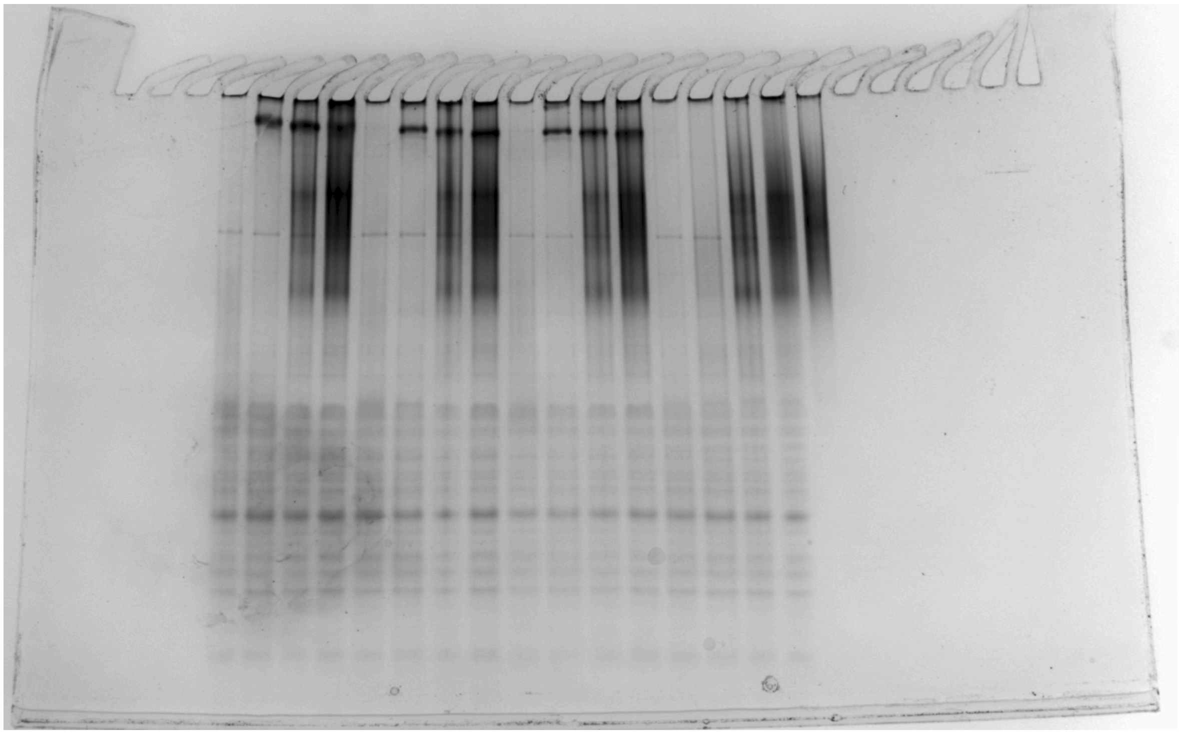

**Supplementary Fig. 2:** Uncropped Native-PAGE image of representative double-mutant assemblies from Extended Data Fig. 1.

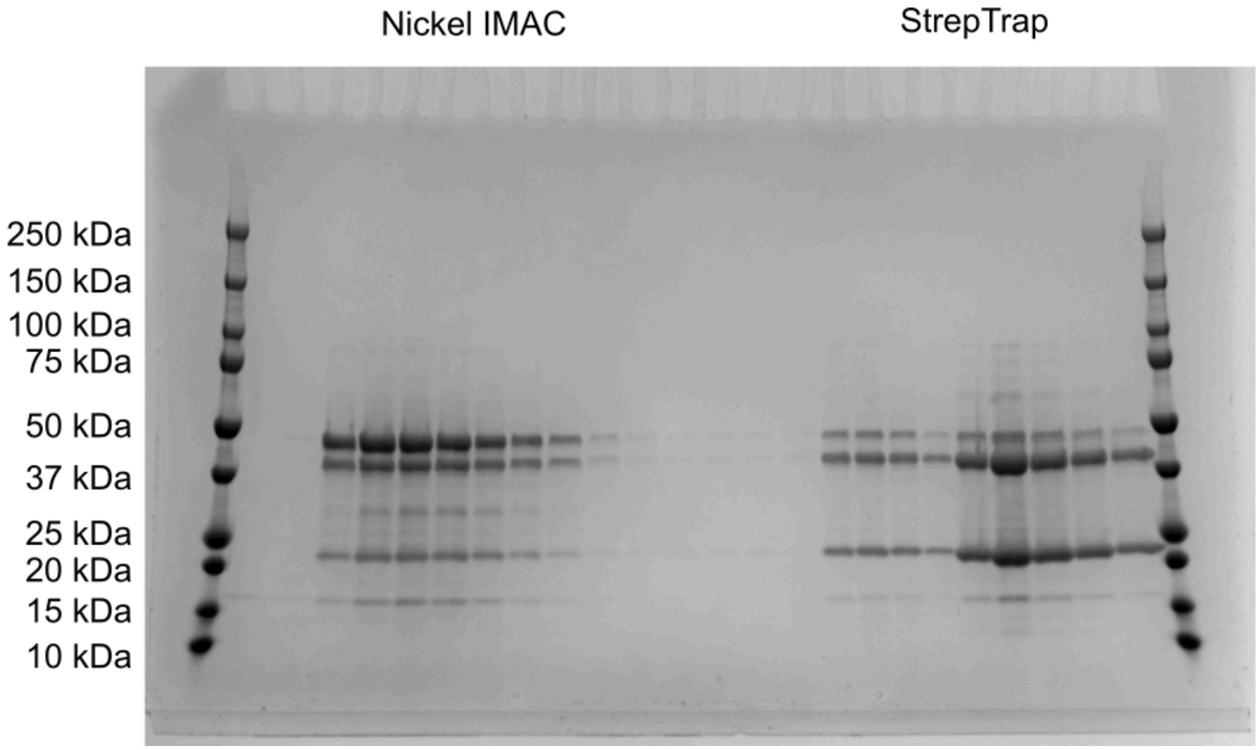


**Supplementary Fig. 3:** Uncropped SDS-PAGE image IMAC and StrepTrap purification gel from Extended Data Fig. 1.

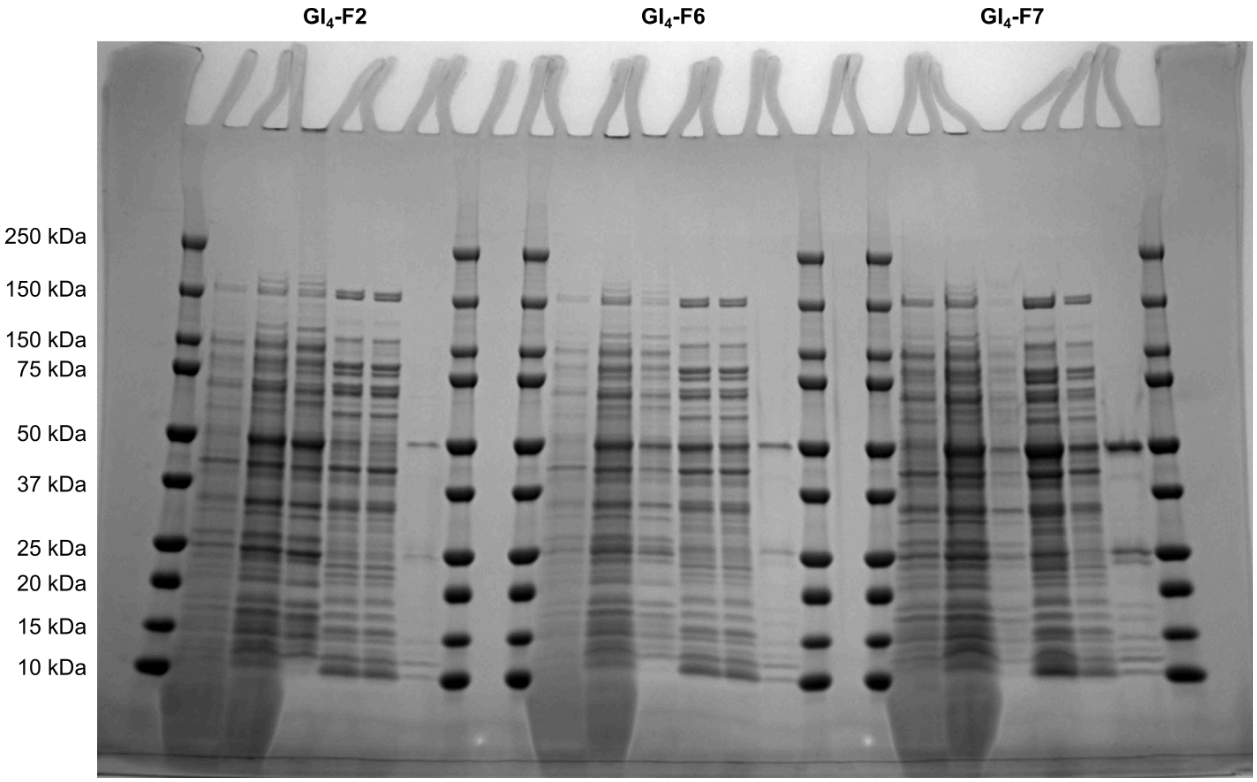


**Supplementary Fig. 4:** Uncropped SDS-PAGE image of nanocage IMAC screening gel from Extended Data Fig. 2.

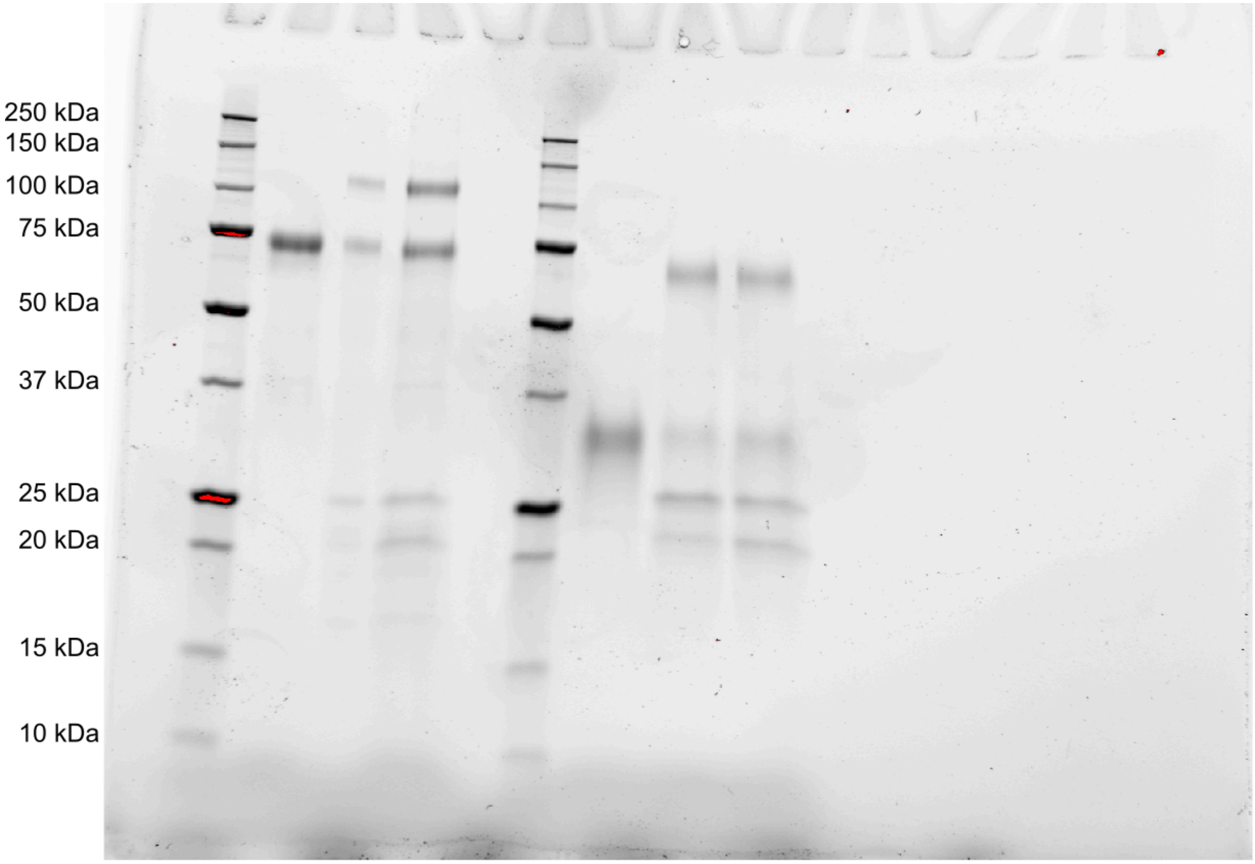


**Supplementary Fig. 5:** Uncropped SDS-PAGE image of antigen-nanocage conjugation from Main Text Fig. 3.

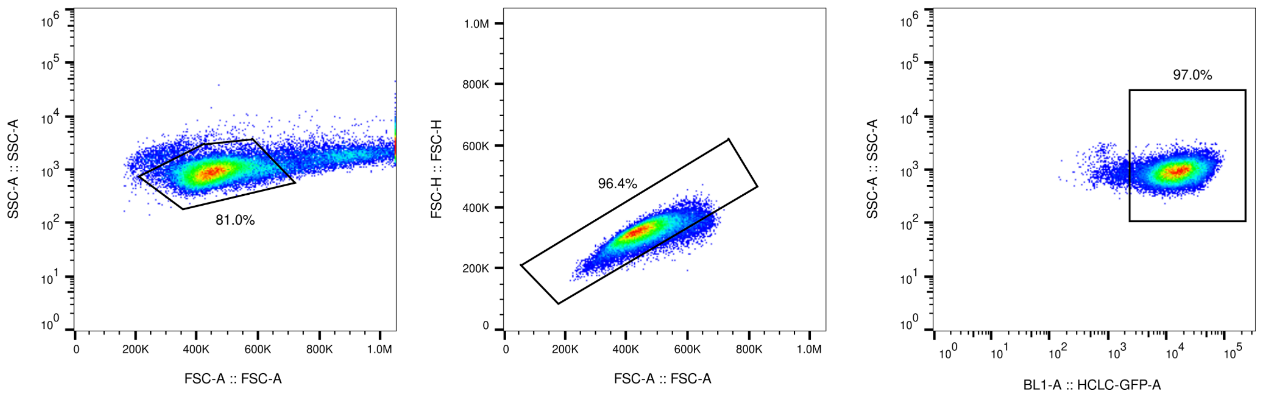


**Supplementary Fig. 6:** Flow cytometry gating strategy for COVA2-15 IgG RAMOS cells. Cells were gated on size, singlets, and GFP reporter for IgG transduced cells before analysis of FuraRed signals.

**Supplementary Table 1: Amino acid sequences for novel proteins used in this study.**

>Pseudosymmetric I53-50A A Chain
MGHHHHHHHHHHGSLQDSEVNQEAKPEVKPEVKPETHINLKVSDGSSEIFFKIKKTTPLRRLMEAFAK
RQGKEMDSLRFLYDGIRIQADQAPEDLDMEDNDIIEAHREQIGGSEKAAKAEEAARKMEELFKKHKIVA
VLRANSVEEAIEKAVAVFAGGVHLIEITFTVPDADTVIKALSVLKEKGAIIGAGTVTSVEQCRKAVESGAEF
**I**VSPHLDEEISQFCKEKGVFYMPGVMTPTEL**Y**KAMKLGHDILKLFPGEVVGPQFVKAMKGPFPNVKFVP
TGGVNLDNVCKWFKAGVLAVGVGKALVKGKPDEVREKAKKFVKKIRGCTE

>Pseudosymmetric I53-50A B Chain
MKMEELFKEHKIVAVLRANSVEEAISKALAVFAGGVHLIEITFTVPDADQVIKELEFLKEAGAIIGAGTVTS
VEQCREAVESGAEFIVS**F**HLDEEISQFCKEEGVFYMPGVMTPTELVKAMKLGHTILKL**V**PGEVVGPQFV
EAMKGPFPNVKFVPTGGVNLDNVCEWFEAGVLAVGVGSALVEGEPAEVAELAIRFVEKIRGCTEGSG
WSHPQFEK

>Pseudosymmetric I53-50A C Chain
MKGEELFTGVVPILVELDGDVNGHKFSVRGEGEGDATNGKLTLKFICTTGKLPVPWPTLVTTLTYGVQC
FARYPDHMKQHDFFKSAMPEGYVQERTISFKDDGTYKTRAEVKFEGDTLVNRIELKGIDFKEDGNILGH
KLEYNFNSHNVYITADKQKNGIKANFKIRHNVEDGSVQLADHYQQNTPIGDGPVLLPDNHYLSTQSVLS
KDPNEKRDHMVLLEFVTAAGITHGMDELYKGGSGGSGGKMEELFKKHKIVAVLRANSVEEAIEKAVAVF
AGGVHLIEITFTVPDADTVIKALSVLKEKGAIIGAGTVTSVEQCRKAVESGAEFIVSP**I**LDEEISQFCKEKG
VFYMPGVMT**F**TELVKAMKLGHTILKLFPGEVVGPQFVKAMKG**A**FPNVKFVPTGGVNLDNVCEWFKAG
VLAVGVGSALVKGTPDEVREKAKAFVEKIRGCTEGSGLNDIFEAQKIEWHE

>Pseudosymmetric GI$_T$ A Chain
MGSHHHHHHGSEKAAKAEEAARKMEELFKEHKIVAVLRANSVEEAKKKALAVFLGGVHLIEITFTVPDA
DTVIKELSFLKEMGAIIGAGTVTSVEQCREAVESGAEFIVSPHLDEEISQFCKEEGVFYMPGVMTPTEL
KAMKLGHTILKLFPGEVVGPQFVEAMKGPFPNVKFVPTGGVNLDNVCEWFEAGVLAVGVGSALVEGT
PVEVAEKAKAFVEKIEGCTE

>Pseudosymmetric GI$_T$ B Chain
MKMEELFKEHKIVAVLRANSVEEAISKALAVFAGGVHLIEITFTVPDADQVIKELEFLKEAGAIIGAGTVTS
VEQCREAVESGAEFIVS**F**HLDEEISQFCKEEGVFYMPGVMTPTELVKAMKLGHTILKL**V**PGEVVGPQFV
EAMKGPFPNVKFVPTGGVNLDNVCEWFEAGVLAVGVGSALVEGEPAEVAELAIRFVEKIRGCTE

>Homotrimeric GI$_T$ BBB
MKMEELFKEHKIVAVLRANSVEEAISKALAVFAGGVHLIEITFTVPDADQVIKELEFLKEAGAIIGAGTVTS
VEQCREAVESGAEFIVSPHLDEEISQFCKEEGVFYMPGVMTPTELVKAMKLGHTILKLFPGEVVGPQFV
EAMKGPFPNVKFVPTGGVNLDNVCEWFEAGVLAVGVGSALVEGEPAEVAELAIRFVEKIRGCTELEHH
HHHH

>Homotrimeric GI$_T$ CCC
MKMEELFKEHKIVAVLRANSREEAIEIALAVFAGGVHLIEITFTVPDADEVIKRLEMLKRAGAIIGAGTVTS
VEQCREAVESGAEFIVSPHLDEEISQFCKEEGVFYMPGVMTPTELVKAMKLGHTILKLFPGEVVGPQFV
EAMKGPFPNVKFVPTGGVNLDNVCEWFEAGVLAVGVGSALVEGKPSEVAEKARRFVKKIRGCTEGSL
EHHHHHH

>RBD-SpyTag
MGILPSPGMPALLSLVSLLSVLLMGCVAETGTRFPNITNLCPFGEVFNATRFASVYAWNRKRISNCVADF
SVLYNSASFSTFKCYGVSPTKLNDLCWTNIYADSFVIRGDEVRQIAPGQTGKIADYNYKLPDDFTGCVIA
WNSNNLDSKVGGNYNYLYRLFRKSNLKPFERDISTEIYQAGSTPCNGVEGFNCYFPLQSYGFQPTNGV
GYQPYRVVVLSFELLHAPATVCGPKKSTGGSGGSGSGGSGGSGSAHIVMVDAYKPTKGGSGGSHHH
HHH

>Homotrimeric GI$_T$ CCC-SpyCatcher
MEELFKEHKIVAVLRANSREEAIEIALAVFAGGVHLIEITFTVPDADEVIKRLEMLKRAGAIIGAGTVTSVEQ
CREAVESGAEFIVSPHLDEEISQFCKEEGVFYMPGVMTPTELVKAMKLGHTILKLFPGEVVGPQFVEAM
KGPFPNVKFVPTGGVNLDNVCEWFEAGVLAVGVGSALVEGKPSEVAEKARRFVKKIRGCTEGSGGSG
GSGGSGAMVDTLSGLSSEQGQSGDMTIEEDSATHIKFSKRDEDGKELAGATMELRDSSGKTISTWISD
GQVKDFYLYPGKYTFVETAAPDGYEVATAITFTVNEQGQVTVNGKATKGDAHILEHHHHHH

---

Appended sequences including SUMO, GFP, SpyTag-, SpyCatcher, and deca- or hexa-histidine, avi-, and strep-tags are underlined.
Pseudosymmetrizing mutations are bold.
